# Rural–urban scaling of age, mortality, crime and property reveals a loss of expected self-similar behaviour

**DOI:** 10.1038/s41598-020-74015-x

**Published:** 2020-10-08

**Authors:** Jack Sutton, Golnaz Shahtahmassebi, Haroldo V. Ribeiro, Quentin S. Hanley

**Affiliations:** 1 School of Science and Technology, Nottingham Trent University, Nottingham, NG11 8NS UK; 2 Departamento de Física, Universidade Estadual de Maringá, Maringá, PR 87020-900 Brazil

**Keywords:** Complex networks, Statistical physics, Cancer, Cardiovascular diseases, Urban ecology

## Abstract

The urban scaling hypothesis has improved our understanding of cities; however, rural areas have been neglected. We investigated rural–urban population density scaling in England and Wales using 67 indicators of crime, mortality, property, and age. Most indicators exhibited segmented scaling about a median critical density of 27 people per hectare. Above the critical density, urban regions preferentially attract young adults (25–40 years) and lose older people (> 45 years). Density scale adjusted metrics (DSAMs) were analysed using hierarchical clustering, networks, and self-organizing maps (SOMs) revealing regional differences and an inverse relationship between excess value of property transactions and a range of preventable mortality (e.g. diabetes, suicide, lung cancer). The most striking finding is that age demographics break the expected self-similarity underlying the urban scaling hypothesis. Urban dynamism is fuelled by preferential attraction of young adults and not a fundamental property of total urban population.

## Introduction

Cities are important drivers of economic and creative human activities^1–4^ and this behavior has long been linked to population^5^. These studies have shown super-linear scaling in urban performance indicators such as patents, GDP, and R&D employment^1–3^. Other less desirable features follow similar scaling such as homicide^4,6^, AIDS cases^1^, and general crime^7,8^. Conversely, there are important economies of scale found in cities in such indicators as road surface and petrol stations^1^. Underpinning this work is the notion of self-similarity leading to behavior which is well approximated by power laws^9^. Modeling this behavior remains an active area of research. These studies have shown that per capita measures are deeply and fundamentally biased in all but the rare metrics which show linear scaling^2,10,11^. This important paradigm shift has not been as widely appreciated beyond the urban scaling community.

Despite the improved understanding of power law scaling in urban regions, linear per capita models remain a cornerstone of many aspects of policy and resource allocation. For example, in the UK regional distribution of health care resources is done via clinical commissioning groups (CCGs). This begins with a per capita allocation which is adjusted for mortality, market forces, and a range of other factors based on nutrition, obesity, smoking, drugs etc^12–14^. The use of scaled metrics provides an opportunity to better understand the taxonomy of health and well-being as well as a host of other metrics. Regional considerations also appear in discussions of economic and social issues in the UK as a north south divide^15–17^. The distribution of population explains some of this, but analysis of regional behaviour relative to scaling law expectations can provide a more definitive view of regional characteristics.

The urban scaling literature has an inherent bias by studying cities and neglecting rural regions. Although the urban population currently exceeds the rural population worldwide^18^, urban areas cover a relatively small amount of the world’s land area and very few studies have looked to see whether cities are fundamentally different from rural regions. Definitions of rural vary. Example definitions of rural areas include: areas which are not urban^19^, areas of low population density and other indicators of rural life^20^, or based on surface urban heat islands^21^. We consider rural and urban to be extremes of a continuum of human environments with population density providing a quantitative metric of position along that continuum. In previous work using data from England and Wales, we found that some metrics follow a single law while others undergo transitions at critical population densities^7^. Metrics undergoing transitions exhibit a range of behaviors: acceleration (e.g. robbery), inhibition (e.g. shoplifting), and collapse (detached housing transactions). The statistical mechanics underlying this behavior remains an unsolved problem, but the existence of critical population densities allows an empirical division between rural and urban. The neglect of rural regions almost certainly neglects their importance assuring the food and material security of heavily urbanized regions.

A consequence of the improved understanding of the effects of scale in indicators is the development of scale adjusted and density scale adjusted metrics^2,7,11^. These were initially developed as indicators of the uniqueness of a particular urban region and used to develop a taxonomy of similar types of cities^2^. The methodology has since been adapted to density scaling of both urban and rural regions where it was used to understand the inter-relationships between crime and property^8^.

Here, we investigated a range of indicators of mortality, crime, property and age throughout England and Wales to determine if mortality behaves similarly to the previous work on crime and property.

## Theory

### Population density scaling

Density scale adjusted metrics^7,8^ are an area normalized approach to scale adjusted metrics^2,11^. Urban scaling uses total population to predict a range of indicator metrics using power laws.

1 $$Y={Y}_{0}{n}^{\beta }$$

where, *Y* is an indicator such as crime or GDP, *Y*_0_ is a pre-exponential factor; *n* is the population density of the region and *β* is a scaling exponent. When looking at both rural and urban regions, density metrics (*y* = *Y*/*A*) and population density (*d* = *n*/*A*) have been found to better predict overall behavior^7,8^ where *A* is the area of a given region.

2 $$y={y}_{0}{d}^{\beta }$$

Similarly to urban scaling, when *β* < 1, the scaling is sub-linear; when *β* = 1, the scaling is linear; and when *β* > 1, the scaling is super-linear. Data is usually fitted to log transformed data to obtain parameters.

3 $$\mathrm{log}y={\mathrm{log}y}_{0}+\beta \mathrm{log}d$$

Empirically, transitions appear at a critical population density, d^*^, for some metrics in the range of 10–70 people per hectare^7^. To account for this, Eq. (3) can be adjusted to allow a segmented fit^7^ at the critical density.

4 $$\mathrm{log}y=\left\{\begin{array}{c}{\mathrm{log}y}_{0}+{\beta }_{L}\mathrm{log}d\\ {\mathrm{log}y}_{1}+{\beta }_{H}\mathrm{log}d\end{array} \begin{array}{c}d<{d}^{*}\\ d \ge {d}^{*}\end{array}\right.$$

In this, *β*_L_ and *y*_0_ are the exponent and pre-exponential factor below the transition; *β*_H_ and *y*_1_ are the exponent and pre-exponential factor above the threshold. For purposes of modelling, the transition point is held to be continuous (e.g. $\mathrm{log}{y}_{1}=\mathrm{log}{y}_{0}+\left({\beta }_{L}-{\beta }_{H}\right)\mathrm{log}{d}^{*}$).

Density scale adjusted metrics (DSAMs)^8^, *z*_*i*_, are the residuals in the fits obtained from the models defined by Eqs. (3) and (4).

5 $${z}_{i}=\mathrm{log}{y}_{i}-\mathrm{log}y$$

A number of issues have been noted when fitting power laws to urban scaling data sets^22^ and particularly when data sets have null values or zeros^23^. In the data considered here, this issue is occasionally severe. Although progress has been made on these problems we note the following: (1) The analysis of scale adjusted metrics^2,8,10^ assumes that the power law fits are an incomplete explanation of the data. Specifically, the approach^2,8,10^ assumes the residuals around a power law fit contain explainable variance and are not random relative to other residuals. (2) Power variance models (e.g. Taylor’s law^22,24^) are good models of the noise in some instances and across limited scales. However, segmented fluctuation scaling occurs at least in the case of crime^24^. (3) Alternatives to power law models have been presented^22,25^ but the extent to which the problems driving their development apply to density scales is unknown. In this context, power law models and the segmented modifications used here remain useful for understanding scale in human systems despite their limitations.

If two arrays of DSAMs corresponding to indicators (*X*, *Y*) over a set of *n* regions are represented by $X=({x}_{1}, {x}_{2}, \dots , {x}_{n})$ and $Y=({y}_{1}, {y}_{2}, \dots , {y}_{n})$, a range of similarity measures (*sm*) can be computed. A region in this context is a defined land area of some size. Here, it represents administrative areas in the UK (unitary authorities, non-metropolitan districts, metropolitan boroughs, and London boroughs) but could be any defined region for which indicator data is available. We considered 6 similarity measures: Pearson correlation (r(*X, Y*)), Spearman correlation ((S(${rg}_{X},{rg}_{Y}$)), Kendall correlation ((K(X,Y)), cosine similarity (c(*X, Y*)), and Jaccard similarities (J(*X, Y*)) to investigate the inter-relationships between the DSAMs.

The matrix of similarity measures (*sm*_*ij*_) generated for each pair of indicator DSAMs (e.g. mortality, property, crime and age) were analyzed by hierarchical clustering based on a distance, ${\delta }_{ij}=\sqrt{2(1-s{m}_{ij})}$.

## Results and discussion

### Overview of regions

England and Wales consist of 348 regions including unitary authorities, non-metropolitan districts, metropolitan boroughs, and London boroughs. The regions ranged in area from 289 ha (City of London, England) up to 518,037 ha (Powys, Wales). Regional populations were from 2158 (Isles of Scilly, England) to 1,070,912 (Birmingham, England) while population density ranged from 0.25 people per hectare (Eden, Cumbria, England) up to 139 p/ha (Islington, England). This covers a range of environments and regions from very rural to highly urban.

### Rural–urban scaling

**Figure 1:**
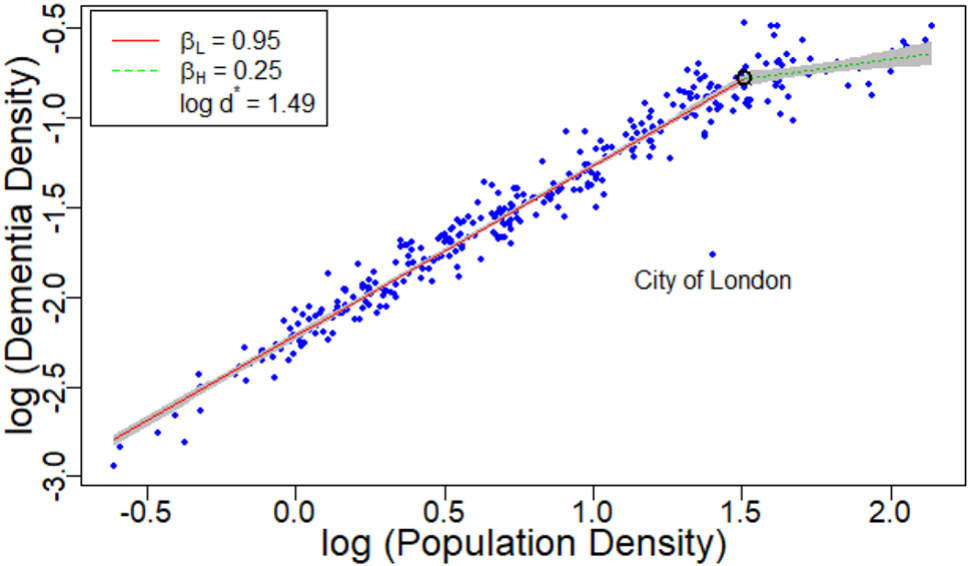
Scaling behavior of mortality from dementia and Alzheimer’s disease. The black circle indicates the position of the critical density. This shows the exceptionally low incidence in the resident population of the City of London. Gray shaded region represents 95% CI.

The density scaling model gave reasonable fits to power laws (e.g. Figs. 1, S1 to S4). Regions did not stand out relative to the scaling laws with the notable exception of the City of London. This region was an obvious outlier in 23 separate metrics and was so extreme that it merits special attention (e.g. Fig. 1). The City of London is a small 289 hectare region within the greater London metropolitan area with a small resident population (7355) and a much larger (> 350,000) daytime population. Scaling laws have been shown to change depending on whether resident or floating population is considered^26^. In our work, many crime indicators gave positive deviations consistent with daytime population. However, dementia mortality and to a lesser extent lung cancer exhibited extreme negative deviation. The generally reduced incidence of dementia in the high population density portion of the scaling plot is intriguing. The trend can be partly explained by a lower proportion of older people. However, the exponents for age and dementia are incommensurate. Dementia mortality decreases to a greater extent than the reduction in older people. This makes the City of London which is nearly a factor of 10 below expectations even more remarkable and future studies of dementia risk should consider a more detailed look at this group of people.

**Figure 2:**
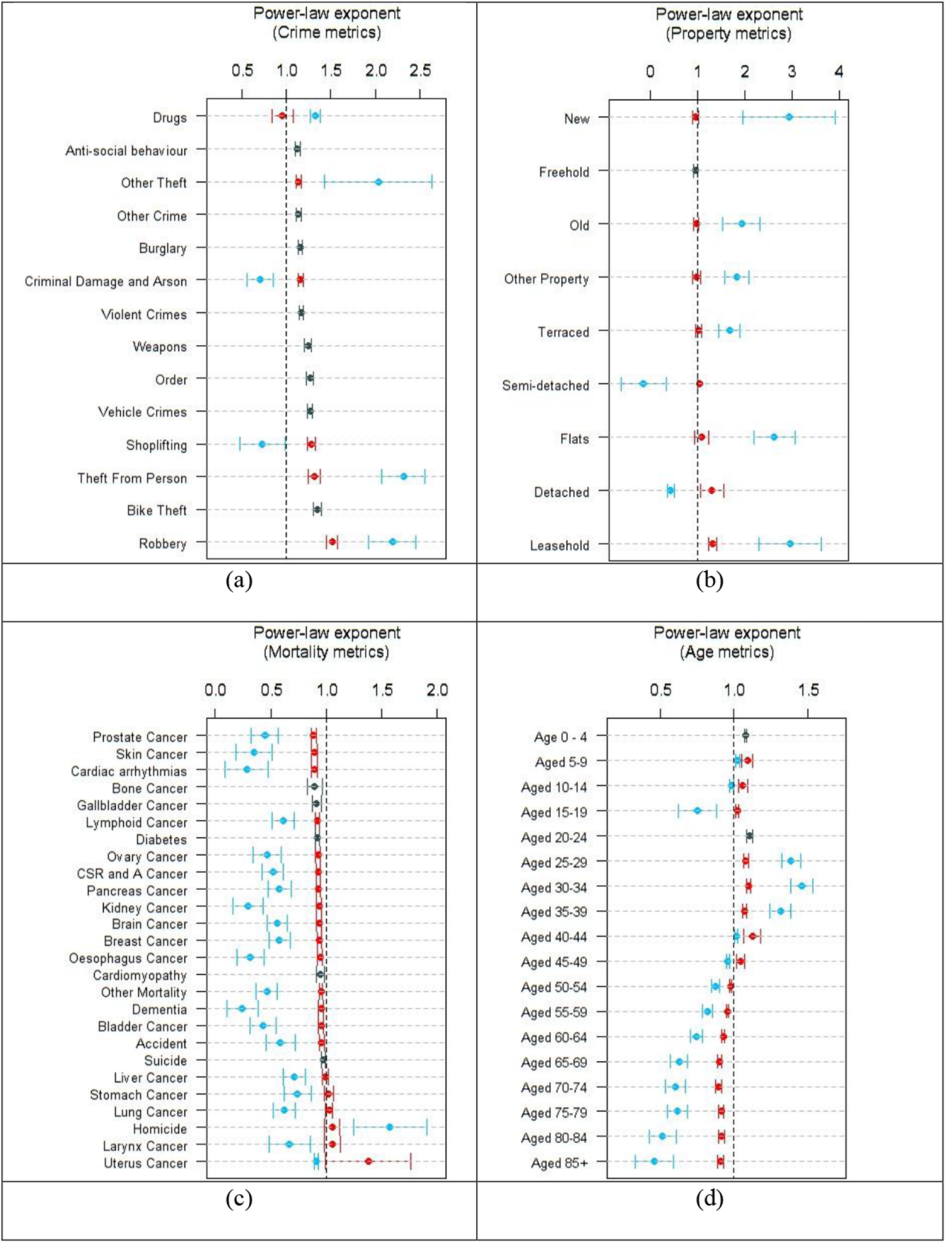
Allometric scaling exponents for crime (**a**), property transactions (**b**), mortality (**c**) and age (**d**) using density metrics. Black symbols indicate exponents for single power-law scaling. Red symbols indicate exponents below d^*^ and blue symbols are for exponents above d^*^. Error bars represent the 95% confidence intervals for $\beta , {\beta }_{L}, {\beta }_{H}$ based on the standard errors of regression. The dotted line represents $\beta =1$.

The density scaling exponents (Figs. 2a,b, S1, S2, S3, and S4; Tables S1 and S2) for crime and property were similar to those observed previously^7^ when parliamentary constituencies were used to define areas. Approximately half of crime metrics followed simple power laws: ASB, Burglary, Vehicle Crime, Violent Crime, Other Crime, Bike Theft, Weapons and Order. The remainder exhibited segmented scaling. Drugs, Other Theft, Theft from the Person and Robbery accelerated while Shoplifting and CD&A were inhibited in high density regions. This heterogeneity of behaviors is a challenge to crime opportunity theory^27,28^ and situational action theories^29,30^. A simple power law suggests uniformly increasing opportunities or criminogenic settings, but critical densities with both acceleration and inhibition require a clearer picture of what these opportunities and criminogenic settings represent. Similarly, the observation of a single relationship defining burglary across all scales challenges the notion of designed environments^31^ for reducing this and the other crime types showing single exponential behavior. The behavior of the eight single power-law crime types is remarkably robust over the entire land area of England and Wales.

Examination of mortality (Figs. 2c, S3, and Table S2) revealed that in rural regions except for 5 types of cancer (liver, stomach, lung, larynx and uterine cancer) and homicide, all mortality indicators exhibited sub-linear to linear scaling. In high density regions, all mortality except homicide was strongly sublinear. The dramatic improvement in mortality can be understood by examining the scaling of age groups.

**Figure 3:**
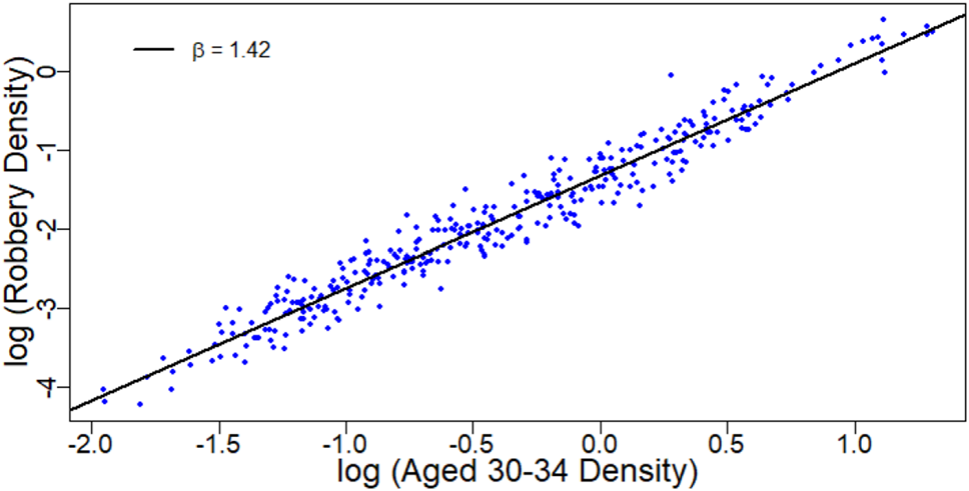
Scaling relationship for Robbery when restricted to the 30 to 34 year age group. The acceleration seen for the general population disappears when age range is restricted.

Population density (Figs. 2d, S4; Table S2) had a profound influence on age demographics. High density regions attract young adults aged 25–39 and people age 45 and over preferentially leave. Although density exponents are not directly comparable to conventional ones^32^, the strength of the super-linear attraction for young people (*β*_H_ = 1.46 for the 30–34 age group) may be sufficient to explain almost all reported super-linear economic indicators^1,2,33^. This can entirely explain the acceleration of Robbery in high density areas (Figs. 3, S1, and Table S2). Age has a strong influence on the exponent for mortality indicators. For example, kidney cancer and dementia show sublinear scaling in high density regions for the general population (Fig. S5, Table S3). When the two oldest age groups are considered, the protective effect of high density remains but is less pronounced. The data is suggestive for homicide having a single scaling exponent when considered using only the 30–34 age group, however, the data is too sparse at high density to reach a robust conclusion with this data set. If this observation holds beyond the UK, it is probably an important underlying mechanism for many effects observed in the urban scaling literature. As a minimum, age groups break the universal self-similarity of the urban scaling hypothesis. Scaling is not constant across age groups.

From a policy perspective, these findings are important. Mortality and health are primarily understood in *per capita* terms. As noted above, UK National Health Service funding is provided through clinical commissioning groups using a formula based primarily on a constant *per capita* cost^12–14^. This *per capita* model may significantly under-estimate the economies of scale in high population density regions and the additional cost associated with delivering effectively to people in rural environments. The extent of the economies of scale are striking and there is a clear rural–urban divide in terms of mortality. The scaling phenomenon explains persistent northern excess mortality in the UK^16^. The regions north of the “north–south” divide have a lower population density and DSAM metrics make clear that the excess mortality is per capita and is commensurate with rural metrics.

### Critical densities

**Figure 4:**
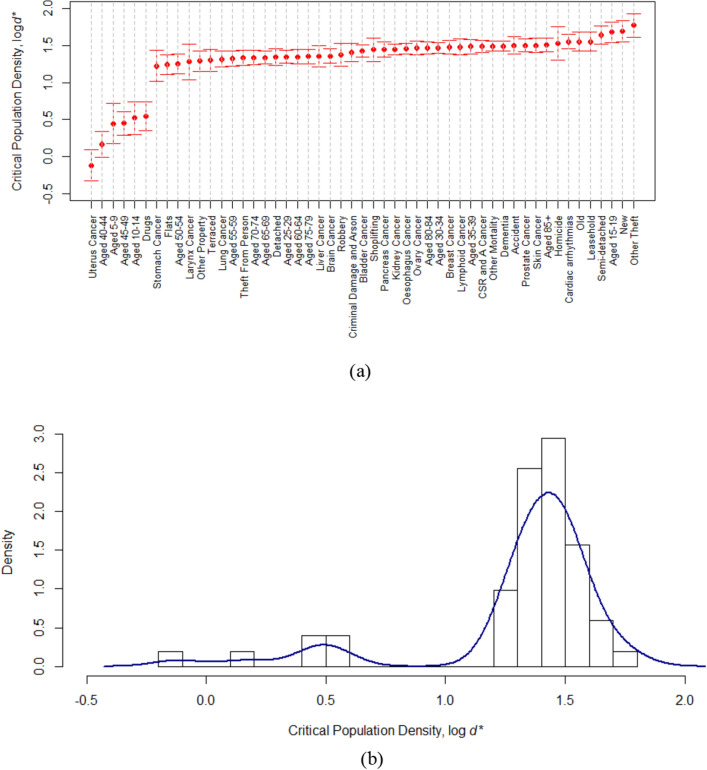
The critical population densities for metrics with segmented scaling. (**a**) Rank order plot of critical densities with the median indicated (dotted line). (**b**) Histogram of critical densities $(\mathrm{log}{d}^{*})$. Error bars represent 95% CI.

Fifty-one out of sixty-seven indicators (6 crime, 8 property, 21 mortality and 16 age) exhibited a critical density (Fig. 4) distributed around a median of 27 p/h. This is similar to the average value of 30 p/h for 19 indicators in our previous work^7^. Although a bimodal density histogram is observed (Fig. 4b), a single distribution dominates. This is remarkable considering they arise from a wide range of indicators including crime, property, mortality and age. The exceptions to the rule include four age groups (aged 5–9, aged 10–14, aged 40–44, aged 45–49). The 40–49 age range is the boundary between the young adults who are super-linearly attracted to high density urban regions and the elderly who preferentially leave. It is likely that were the age ranges defined differently no critical point would be observed and the change in exponent around the critical values for all four is relatively small. Without these transitional age groups, only two exceptions remain. For the 45 indicators with critical densities in the same distribution, there is currently no explanation. There is no explanation for why mortality, crime, and property scaling pivots around a critical density. Age group behavior is important, but there is no explanation for the preferential attraction of young people to regions above a critical density. The critical density appears robustly near 27 p/h, but the reason it appears at that scale is unclear. The physics of percolation transitions^34–36^ may offer solutions, but a unifying statistical mechanics remains to be found which predicts a transition in human behavior (crime), health (mortality), economics (property transaction values), and age demographics at a critical density remains to be found.

### Correlation and hierarchical clustering of DSAMs by category

**Figure 5:**
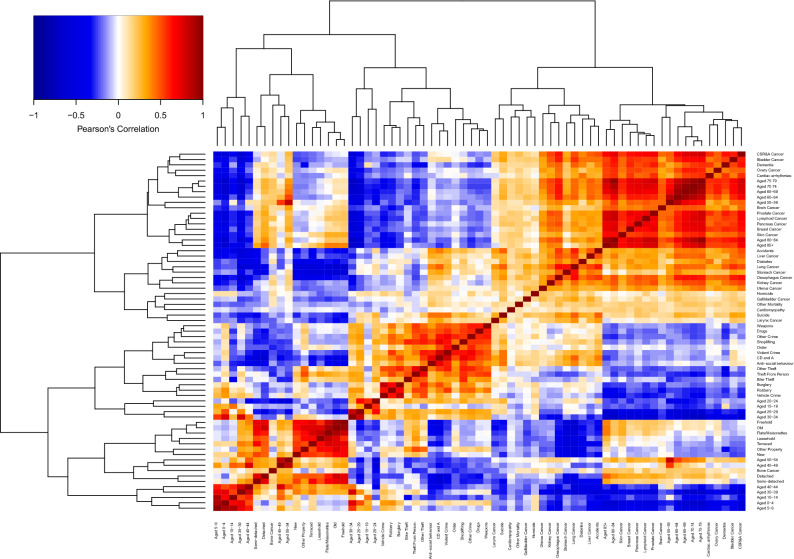
Indicator clustering and heatmap. The color of the heatmap refers to the relationship between every pair of DSAMs (*i* and *j*) by evaluating the Pearson correlation coefficient $({\rho }_{i,j}$). The red indicates positive correlation and blue indicates negative correlation. The darker the shade the stronger the correlation. The upper and left side panels are the dendrograms constructed via the hierarchical clustering algorithm.

Correlation analysis and hierarchical clustering of DSAMs (Fig. 5) showed a tendency for crime, property, and mortality to be positively correlated and cluster together with other members of their class. As examples, most mortality types were positively correlated and clustered with all other mortality types (Fig. S6) with many correlations above 0.5 and values reaching 0.72 (e.g. Fig. S7: Lymphoid Cancer vs. Prostate Cancer). The exceptions were bone cancer, larynx cancer, and homicide. All crime types were positively correlated (Fig. S8) and clustered (Fig. 5) with all other types of crime. All property types were positively correlated (Fig. S9) and clustered (Fig. 5) with all other property types with some very strong correlations (e.g. Freehold vs. Old properties had Pearson correlation = 0.95 (Fig. S10)).

Age did not follow this pattern. Age groups were highly stratified with children and old people anti-correlated (Fig. S11). Different age ranges clustered with different indicator classes. Young people (15–34) clustered with crime. Older people (55 and above) clustered with mortality except for bone cancer. The very young (0–14) and middle-aged people (35–54) clustered with property. Most correlations in the first two clusters were positive, while most property indicators were anti-correlated with children aged 0–14.

The most striking finding is the division of the bulk of mortality indicators into two groups. One group clustered with the elderly and tended to have positive correlation with certain types of property DSAMs. The other group, nearly all of which are to some degree preventable (Accidents, Liver Cancer, Diabetes, Lung Cancer, Stomach Cancer, Oesophagus Cancer, Kidney cancer, Uterus Cancer, Homicide, Suicide and Larynx Cancer) had nearly universal anti-correlation with property DSAMs (c.f. Fig. S12, Flat vs. Lung Cancer DSAMs). The extent to which the magnitude of property transaction value exceeds scaling expectations protects against a wide range of mortality from preventable conditions ranging from homicide to uterine cancer. These conclusions were generally reinforced by all correlation measures (Figs. S13 and S14). The similarity measures were less informative (Figs. S15 and S16).

**Figure 6:**
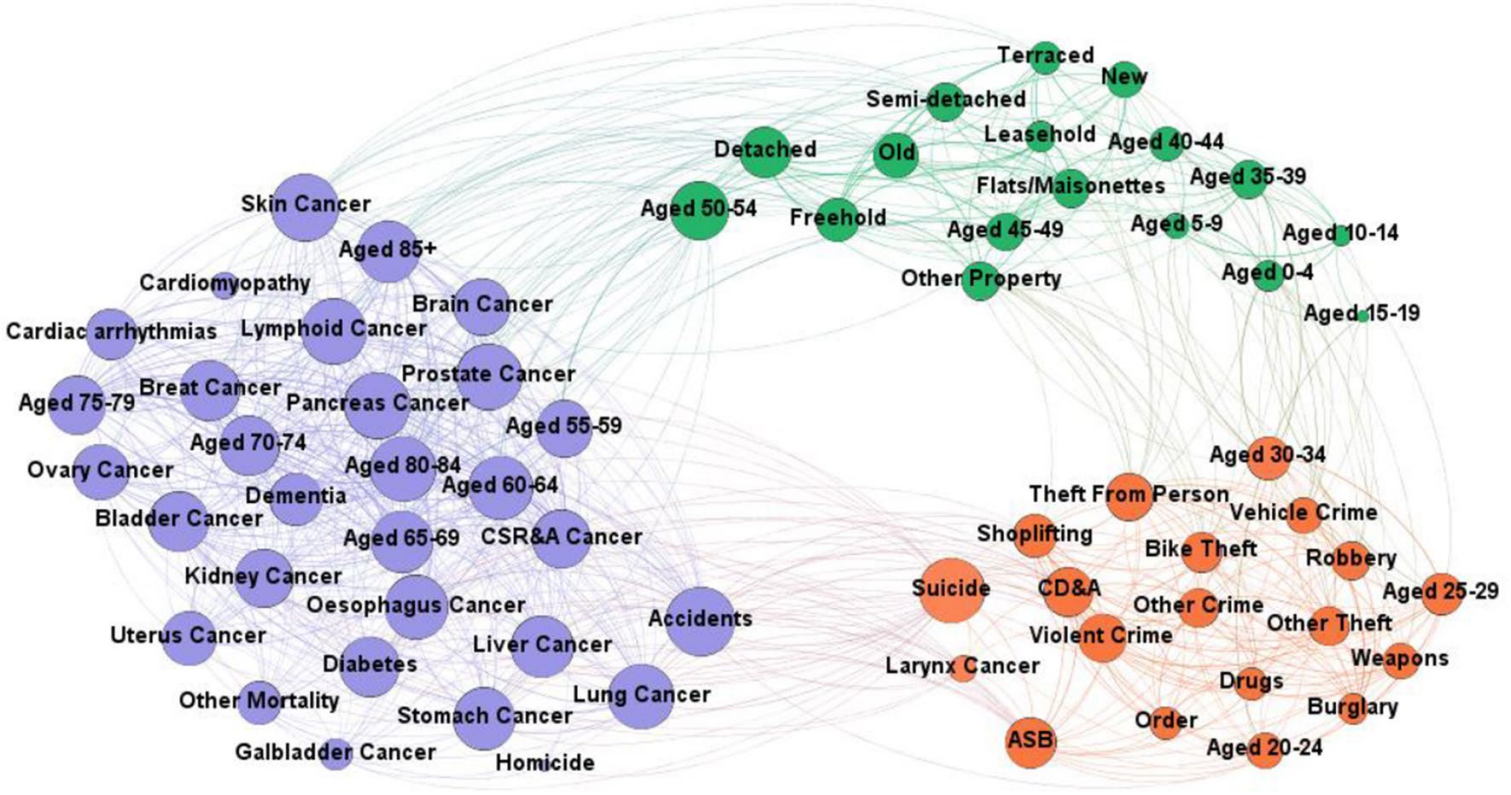
Network of statistically significant positively correlated DSAMs. Network representation for the positive connections among crime, property, mortality and age DSAMs. The colors indicate network module and the edge thicknesses are proportional to the correlation. Node sizes are proportional to their degrees.

A limitation of the heatmap and clustering (Fig. 5) is the pairwise structure which does not display the significance of the correlations. A network accounting for this was created by bootstrapping the Pearson correlation with 2000 replications for every pair of metrics to identify correlations significant at 99% confidence. The resulting network (Fig. 6) has 66 nodes including all metrics except bone cancer which had no statistically significant correlations. There were 784 significant connections out of 2211 possible and the optimal modularity score (0.472) partitioned the network into 3 communities very similar to the clusters in the indicator heatmap (Fig. 4).

Specifically, the network analysis found three modules containing: the elderly and mortality; children, middle-aged people and property; and young adults and crime. There were only two exceptions to this pattern, suicide and cancer of the larynx, which clustered with young adults and crime. These two were also most closely related to each other in the clustering analysis. Cancer of the larynx has long been associated with alcohol^37^ and smoking^38^ and preventative measures beyond cessation are limited. The association with suicide as well as the positive correlations of cancer of the larynx and suicide with ASB, CD&A, violence, accidents, diabetes, liver and lung cancers suggests health care delivery focusing on mental health^39,40^, alcohol^41^, and community safety may be beneficial for this group. Considering these types of mortality as long term responses to violence, stress, and mental illness could lead to more efficient prevention strategies.

### Analysis of DSAMs by region

**Figure 7:**
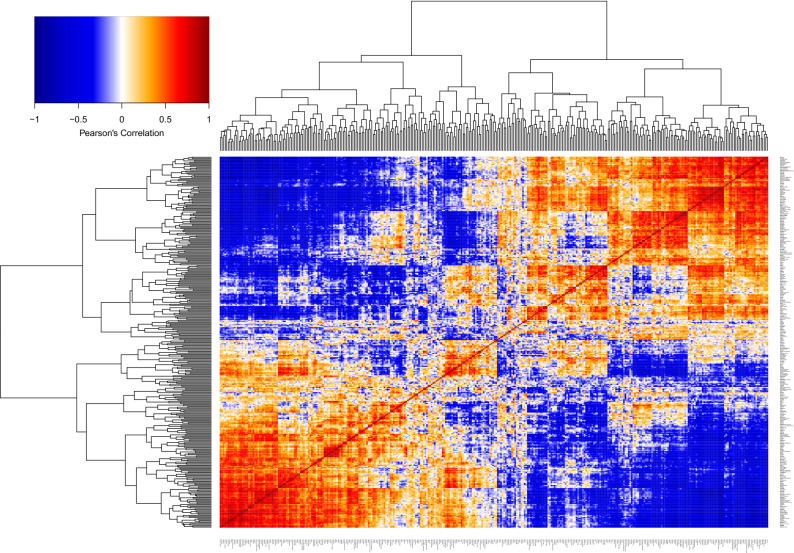
Regional heatmap and clustering. Format and color coding are the same as Fig. 4. The hierarchical clustering roughly divides England and Wales into two regions. A geomap of these two regions appears as Fig. S17.

To understand regional behavior the clustering and correlation analysis was repeated on the transpose of the matrix of DSAMs such that it was presented by region rather than indicator (Fig. 7). Although heterogeneity is seen, broadly two clusters appear with universal anti-correlation at the extreme ends. The two extreme ends (e.g. Stoke-on-Trent vs. Bromley) live in nearly opposite worlds. If crime and mortality are above expectation in one it is below in the other. A geomap of the two clusters (Fig. S17) divided North England, Wales and the Midlands from Southern England with some exceptions.

### Self-organizing maps

**Figure 8:**
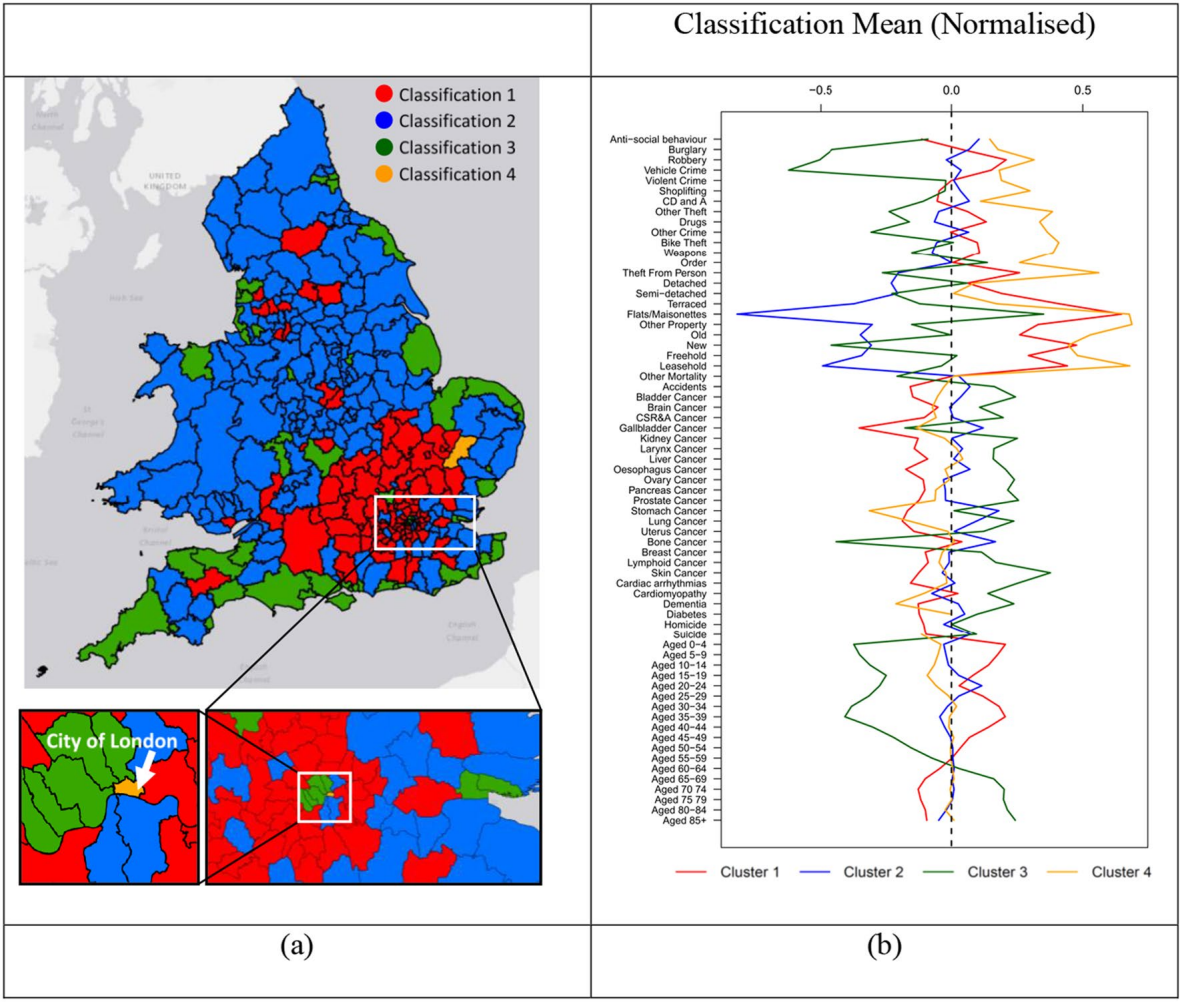
Map of England and Wales coded by self-organizing clusters. (**a**) The map includes all 348 Unitary Authorities, non-metropolitan districts, metropolitan boroughs and London boroughs colored by cluster. (**b**) The mean DSAMs for the 4 regions are shown after normalization to facilitate comparison. Un-normalized versions are available as Fig. S20. Map was created using R version (3.6.2) (https://www.r-project.org/) running under R-Studio (Version 1.2.5019) (https://rstudio.com/).

The simple geomap (Fig. S17) did not provide sufficient understanding of regional heterogeneity apparent in the cluster analysis. Regions are also affected by age demographics and their importance needs to be understood better. To explore regional behavior, the 348 regions were distributed onto an 8 by 8 hexagonal self-organizing map (Fig. S18). After 350 iterations convergence was reached (Fig. S19) with 4 clusters containing 2, 95, 190 and 61 regions which were colored orange, red, blue and green, respectively (Fig. 8).

The four clusters represent: (i) 61 mostly coastal areas (green) with a few more urban inland regions consisting of St. Helens, Stoke-on-Trent, Wyre Forest, Malvern Hills, Strafford-on-Avon, Dacorum, Ipswich, Kensington and Chelsea, Hammersmith and Fulham, City of Westminster, Islington and Camden); (ii) 2 regions (orange) including the City of London and St. Edmundsbury; (iii) 95 regions (red) mostly within the south of England (exceptions are: Richmondshire, Leeds, Bradford, Preston, Chorley, Blackburn with Darwen, Rosendale, Trafford, Manchester); and (iv) 190 more rural regions (blue) primarily in the North of England and Wales.

A key feature of the primarily coastal grouping was excess mortality and a more elderly demographic (people aged 60 +). The City of London and St. Edmundsbury were exceptional in comparison to the other clusters. They exhibit extremely low DSAMs for nearly all mortality types and high property and crime DSAMs driven by the City of London and to a lesser extent by St. Edmundsbury. Characterizing this cluster is aided by a plot of St. Edmundsbury Vs. City of London (Fig. S21) which shows the similarity to be related to high crime and property DSAMs and low mortality. It is important to note that the SOM classification is not based on correlation. Thus, the large group of more neutral indicators form part of the overall picture. The cluster primarily in the South of England is characterized by low mortality, a younger age demographic, and high property DSAMs. The remaining cluster (blue) represents most of the area of England and Wales. These are generally average for age, crime, and mortality with below expectation property DSAMs.

## Conclusion

This study represents an advance in our understanding of scaling behavior while challenging the urban scaling hypothesis. It supports the general concept of scaling by making clear the problems of *per capita* models when applied to health outcomes. However, incommensurate scaling in different age demographics is a challenge. The scaling hypothesis considers all people as equal participants in the acceleration of life in cities. The data here shows that much of that acceleration depends on the ability of urban regions to attract young adults. Observed urban scaling is a consequence of separate scale related processes that define the behavior of specific age demographics around a critical transition in human behavior at the rural and urban boundary.

The consequences of this are great. There have now been many studies making clear that linear *per capita* measures are biased^1,4,7,10,11^. The current study is the first to extend this to mortality from non-transmissible diseases and age demographics. Epidemiologists studying excess death need to understand the bias of *per capita* models. For example, the observed northern excess mortality in the UK^16^ reflects mortality at low population density rather than north–south division. The north mostly falls into a single category (the blue region of Fig. 7) and this region does not have exceptional mortality for the population densities. Policy makers need to understand the limitations of linear *per capita* models. In terms of mortality outcomes, there are large cumulative economies of scale between the most rural and the highest density urban regions. This is a consequence of scale related changes in age groups, to scaling behavior across all population densities, and conditions where high density areas provide protection (e.g. dementia). Within this context, health care resourcing is skewed in favor of population dense regions.

The robust rural–urban division near 25–30 p/h makes clear that ignoring rural regions is a missed opportunity for researchers studying urban systems. The existence of a rural–urban boundary justifies the study of cities while providing a clearer comparison against which claims about urban areas can be made. The lack of a clear explanation for critical densities and why they appear in such a consistent place is an important unsolved problem.

The success of DSAMs and related methodologies^2,8,10,11^ makes clear that any set of scaling laws provides an incomplete picture of both rural and urban landscapes. Although they may appear to be, the residuals are not randomly distributed around the scaling law whatever the model. They are extensively correlated and reveal persistent structure and regional variation.

## Materials and methods

### Data sets

Data on mortality and age were provided by NOMIS (https://www.nomisweb.co.uk) a database service for labour market statistics run by the University of Durham on behalf of the UK Office of National Statistics. To anonymise the mortality data, NOMIS sets values ≤ 2 to 0 and values of 3 and 4 to 5 causing some distortion of low values and rare events. The age demographic data is model adjusted for a particular year based on the most recent census. Population, land area, crime and property information were obtained from the UK Home Office and Land Registry via UKCrimeStats (https://www.ukcrimestats.com) which provides alignment of public data sets using geographic shape files obtained from the Ordnance Survey Boundary Line dataset. Data covering the period from 2013–2017 were captured on 20/03/2019. A total of 67 indicators were obtained (Table 1) and are available as S1 Dataset.

**Table 1:** Comprehensive list of indicators studied. Sixty-seven indicators were studied: 14 indicators of crime, 9 indicators of property, 26 indicators of mortality and 18 indicators of age.

**Crime types**		
Anti-social behaviour (ASB)	Bike theft	Burglary
Criminal damage and Arson (CD & A)	Drugs	Order
Other crime	Other theft	Robbery
Shoplifting	Theft from person	Vehicle crime
Violent crime	Weapons	
**Property types**		
Detached	Flats	Freehold
Leasehold	New	Old
Semi-detached	Terraced	Other Property
**Mortality types**		
Accidents	Bladder cancer	Brain cancer
Colon, sigmoid, rectum and anus cancer (CSR&A)	Gallbladder cancer	Kidney cancer
Larynx cancer	Liver cancer	Oesophagus cancer
Ovary cancer	Pancreas cancer	Prostate cancer
Stomach cancer	Lung cancer	Uterus cancer
Bone cancer	Breast cancer	Lymphoid cancer
Skin cancer	Cardiac arrhythmias	Cardiomyopathy
Dementia	Diabetes	Suicide
Homicide	Other mortality	
**Age categories**		
Aged 0–4	Aged 5–9	Aged 10–14
Aged 15–19	Aged 20–24	Aged 25–29
Aged 30–34	Aged 35–39	Aged 40–44
Aged 45–49	Aged 50–54	Aged 55–59
Aged 60–64	Aged 65–69	Aged 70–74
Aged 75–79	Aged 80–84	Aged 85 +

### Networks

Previous studies of crime and property found that DSAMs show extensive correlations and form modular networks^8^. The network representation $N=(V, E)$ consists of nodes $V=\left\{{v}_{1}, {v}_{2}, \dots , {v}_{n}\right\}$ and edges $E=\left\{{e}_{1}, {e}_{2}, \dots , {e}_{m}\right\}$. Here, nodes are indicators (e.g. Burglary, Suicide, etc.) and edges between them indicate two indicator metrics (*i* and *j*) with significant positive Pearson’s correlation, ${\rho }_{i,j}$ , between their corresponding DSAMs (edges weighted by ${\rho }_{i,j}$). The Pearson correlation was selected based on our previous work^7^. Indicators in networks were clustered by modularity optimization to detect community structure^42–45^.

### Self-organizing maps

A self-organizing map is an iterative approach to representing high dimensional datasets in a low dimensional space^46,47^ using a pre-defined array of nodes, *m*, arranged in a “grid-like” structure. We selected an 8 × 8 hexagonal array of nodes initialized to a random weight ${w}_{ij}$ in the interval [0, 1]. This array was the largest *n* × *n* array without empty nodes^48^. The nodes were then updated after introducing each regional DSAMs input vector ${x}_{1}, \dots , {x}_{n}$ at iteration $t$. The distance, $D\left(j\right),$ was obtained by calculating the Euclidean distance between the input vector and weight vector for all units such that:

6 $$D\left(j\right)=\sum_{i=1}^{n}\sum_{j=1}^{m}{\left({x}_{i}-{w}_{ij}\right)}^{2}$$

The input vector (regional DSAMs) was assigned to the unit index $j$ that has the minimum Euclidean distance. The weight vector ${w}_{ij}$ is updated on the “winning” unit $j$ after each iteration such that:

7 $${w}_{ij}\left(t+1\right)={w}_{ij}\left(t\right)+\alpha \left(x(t)-{w}_{ij}(t)\right)$$

where $x\left(t\right)$ is the input vector’s instance at iteration *t*, ${w}_{ij}\left(t\right)$ is the old weight, ${w}_{ij}\left(t+1\right)$ is the new weight and $\alpha$ is the learning rate in the interval [0, 1], which decreases with $t$, to ensure the network converges. After the learning phase, all observations (i.e. regions) are positioned into a node within the map. If two or more observations are positioned within the same node this shows similarity.

The nodes were clustered by the standardized gap statistic^49^,

8 $${Gap}_{n}\left(k\right)={E}_{n}^{*}\left\{\mathrm{log}\left({W}_{k}\right)\right\}-\mathrm{log}\left({W}_{k}\right)$$

where $k$ is the number of clusters, ${W}_{k}$ is the pooled within-cluster sum of squares around the cluster means and ${E}_{n}^{*}$ denotes expectation under a sample of size *n* from the reference distribution^49^ usually a uniform distribution (i.e. a distribution with no obvious clustering). An estimate of ${E}_{n}^{*}\left\{\mathrm{log}\left({W}_{k}\right)\right\}$, is obtained by simulating B samples of $\mathrm{log}\left({w}_{k}^{*}\right)$ each of size *n* generated from a Monte Carlo sample ${X}_{1}^{*}, . . . , {X}_{n}^{*}$ drawn from the reference distribution. In each case, ${E}_{n}^{*}\left\{\mathrm{log}\left({W}_{k}\right)\right\}$ is an average of B samples of $\mathrm{log}\left({w}_{k}^{*}\right)$. Therefore, assuming the reference distribution is a uniform distribution, a large gap statistic means that the clustering structure does not resemble uniformly distributed observations. Thus, the optimal number of clusters *k* occurs when $Gap\left(k\right)\ge Gap\left(k+1\right)-{s}_{k+1}$. Here, ${s}_{k}$ is the simulation error in ${E}_{n}^{*}\left\{\mathrm{log}\left({W}_{k}\right)\right\}$.

### Data analysis

The data were analyzed using the statistical software R version (3.6.2)^50^ with the Segmented (1.1–0)^51–54^, proxy (0.4–2.4)^55^, boot (1.3–2.4)^56,57^, kohonen (3.0.1)^58,59^, and factoextra (1.0.6)^60^, moments (0.14)^61^, gplots (3.0.3)^62^, ggplot2 (3.3.1)^63^, car (3.0–8)^64^, nortest (1.0–4)^65^, RColorbrewer (1.1–2)^66^, NbClust (3.0)^67^, tidyverse (1.3.0)^68^, cowplot (1.0.0)^69^, psych (1.9.12.31)^70^, sf (0.8–1)^71^, raster (3.0–12)^72^, dplyr (0.8.3)^73^, spData (0.3.3)^74^, tmap (2.3–2)^75^, leaflet (2.0.3)^76^, mapview (2.7.0)^77^, shiny (1.4.0.2)^78^, and png (0.1–7)^79^ packages. The data were log transformed and analyzed by piecewise regression. The Davies test was used to test the significance of any changes of slope with a 99% confidence level set for inclusion of a second segment. The Davies test and Akaike (AIC) and Bayesian (BIC) information criteria were used to select single and double exponential models. The residuals from the selected model were computed and used directly as DSAMs. Correlation and similarity measures were investigated including Pearson, Spearman and Kendall correlation, cosine similarity, and Jacquard distance using the proxy package computed in a pairwise manner for all indicator metrics and regions. The Pearson correlation and uncertainties were bootstrapped using the boot package to find significant connections at 95% confidence. The obtained connections for both the indicators and the regions are used to form positive and negative networks. The networks were constructed using Gephi version (0.9.2)^80^. The self-organizing maps (SOM) were constructed using the kohonen package to investigate regional characteristics. A range of clustering methods were deployed on the SOM using the package factoextra to find an optimal number of clusters. These clusters are represented in the regional maps.

## Supplementary information

Supplementary file1

Supplementary file2

Supplementary file3

Supplementary file4

Supplementary file5

Supplementary file6

## Data Availability

All data generated or analysed during this study are included in this published article (and its supplementary information files). This data was compiled from a range of publicly available sources as noted in the manuscript. These are provided as the Following files: S1_data_raw.csv, S1_data_densities.csv, S1_data_cluster_means.csv, and S1_data_residuals.csv.

## Code availability

We have also provided a set of R-scripts as supplementary information. This has been provided as S1_maincode_rev1.R.
