# Supplementary material for: Rural–urban scaling of age, mortality, crime and property reveals a loss of expected self-similar behaviour
Source: Sci Rep. 2020 Oct 8;10:16863. doi: 10.1038/s41598-020-74015-x (PMC7545192; doi:10.1038/s41598-020-74015-x)
Supplement: Supplementary file 6 — Supplementary file6 [file 41598_2020_74015_MOESM6_ESM.docx]

### Supplementary Information for: Rural-Urban Scaling of Age, Mortality, Crime and Property Reveals a Loss of Expected Self-Similar Behaviour

Jack Sutton,^*^ Golnaz Shahtahmassebi,^*^ Haroldo V. Ribeiro,*^‡^* and Quentin S. Hanley^*^

**School of Science and Technology*

*Nottingham Trent University*

*Nottingham NG11 8NS*

*United Kingdom*

*‡Departamento de Física,*

*Universidade Estadual de Maringá,*

*Maringá, PR 87020-900,*

*Brazil*

**Corresponding author:** Q. S. Hanley
Email:Quentin.hanley@ntu.ac.uk

**Similarity measures.**

The Pearson correlation between two sets of DSAMs is the covariance divided by the product of the standard deviations:

$$\rho\left( X,Y \right)=\frac{Cov(X,Y)}{\sigma_{X}\sigma_{Y}}$$

Eq S1

The Spearman correlation is the Pearson correlation coefficient between the rank variables. Thus, the DSAMs $X_{i}, Y_{i}$ are converted to ${rg}_{X_{i}},{rg}_{Y_{i}}$ and the covariance divided by the product of the standard deviations:

$$S\left( {rg}_{X},{rg}_{Y} \right)=\frac{Cov({rg}_{X},{rg}_{Y})}{\sigma_{{rg}_{X}}\sigma_{{rg}_{Y}}}$$

Eq S2

The Kendall correlation between DSAMs is the difference between concordant pairs and discordant pairs divided by the binomial coefficient for the number of ways to choose two items from $n$ items:

$$K\left( X,Y \right)=\frac{n_{c}-n_{d}}{\left( \begin{matrix} n \\ 2 \end{matrix} \right)}$$

Eq S3

The cosine similarity between DSAMs is the dot product of the two vectors divided by the product of the two vectors’ magnitude:

$$c\left( X,Y \right)=cos(\theta)=\frac{\boldsymbol{X\cdot Y}}{\left\| \boldsymbol{X} \right\|\left\| \boldsymbol{Y} \right\|}$$

Eq S4

The Jaccard similarity which is a measure of dissimilarity between DSAMs is the size of the intersection divided by the size of the union:

$$J\left( X,Y \right)=\frac{\left| X\cap Y \right|}{\left| X\cup Y \right|}=\frac{\left| X\cap Y \right|}{\left| X \right|+\left| Y \right|-\left| X\cap Y \right|}$$

Eq S5

**S1 Dataset. Data employed in this study.** Data covering the period from 2013-2017 were captured on 20/03/2019.

| Log(Indicator Density) | 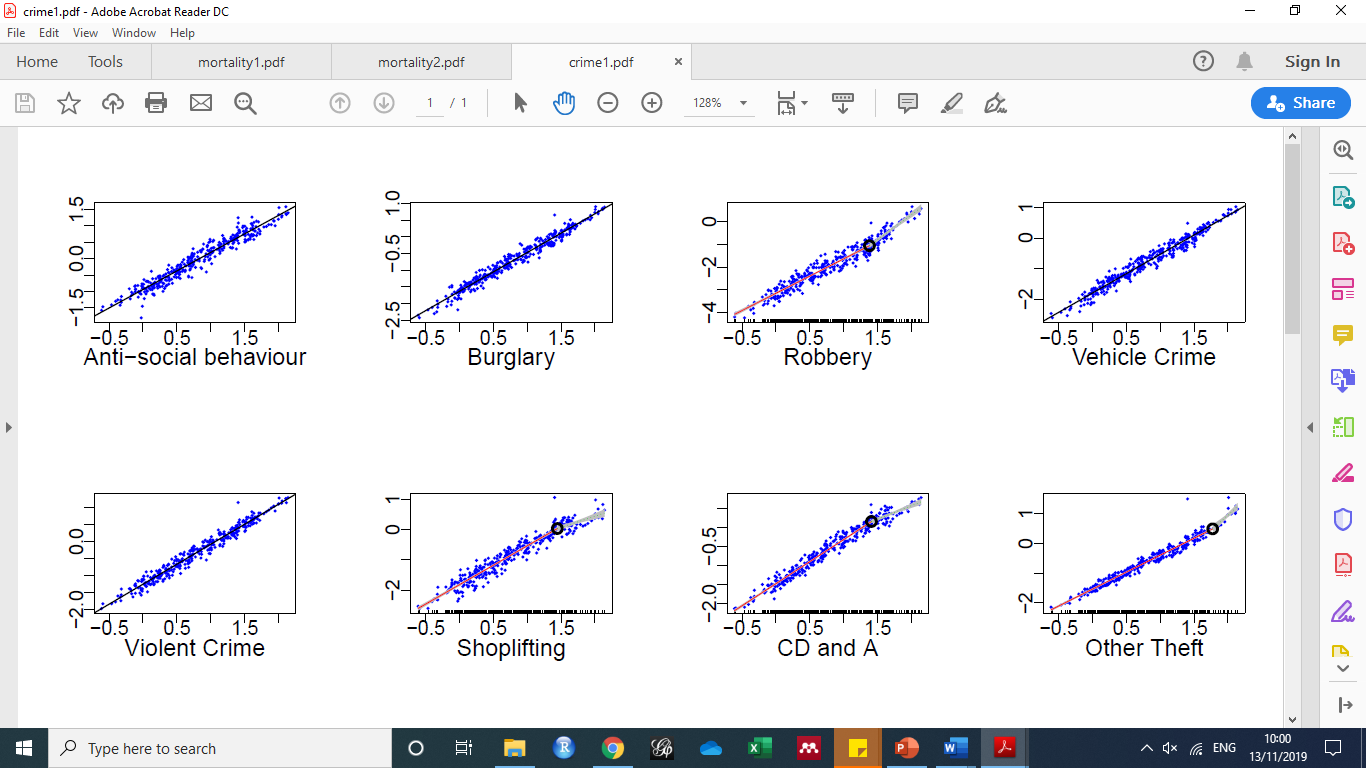 |
| --- | --- |
|  | 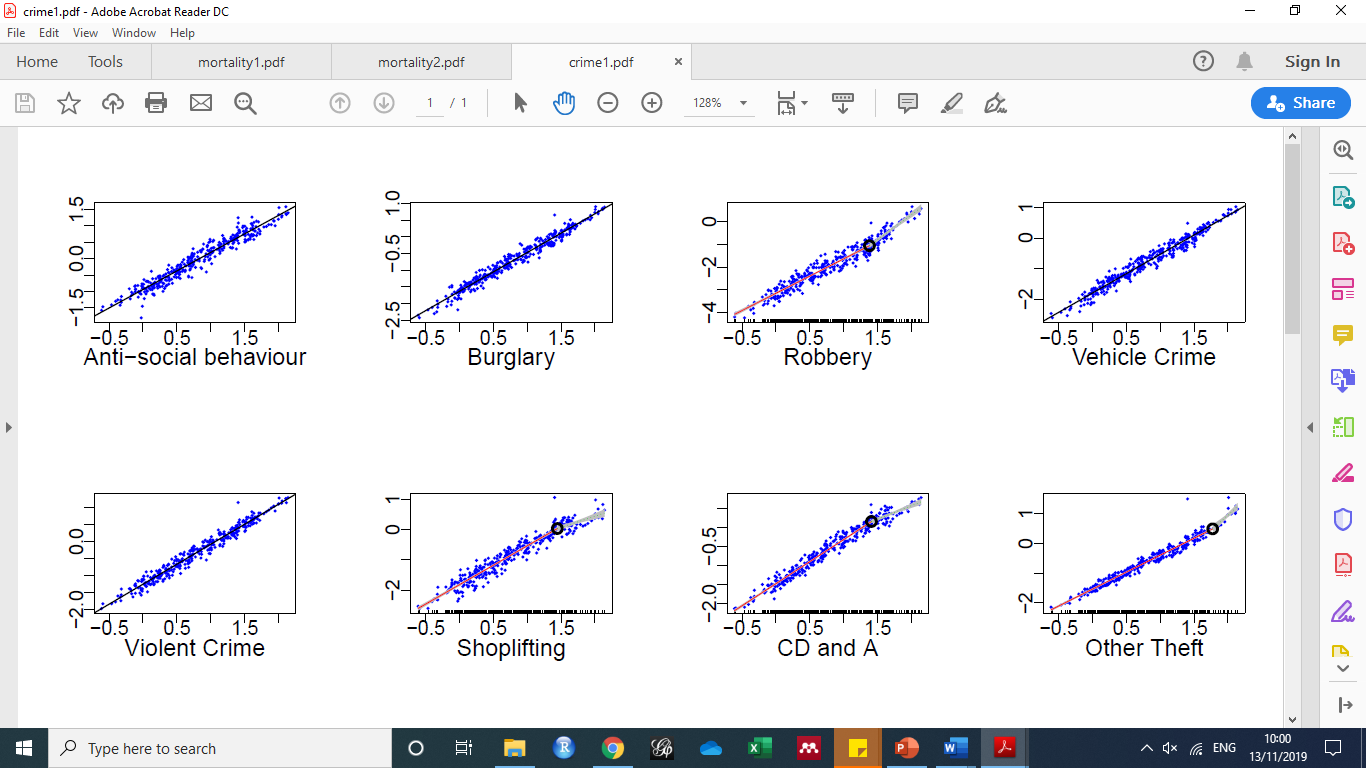 |
|  | 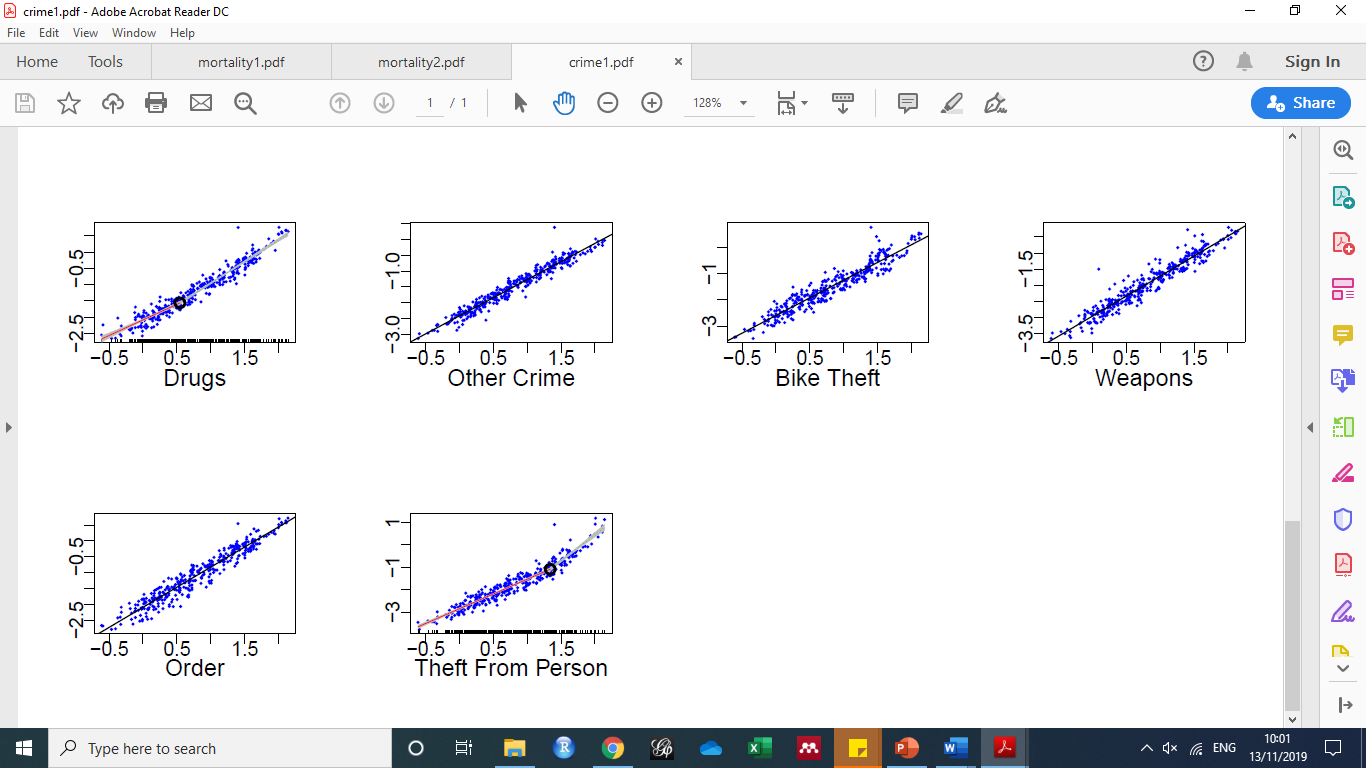 |
|  | 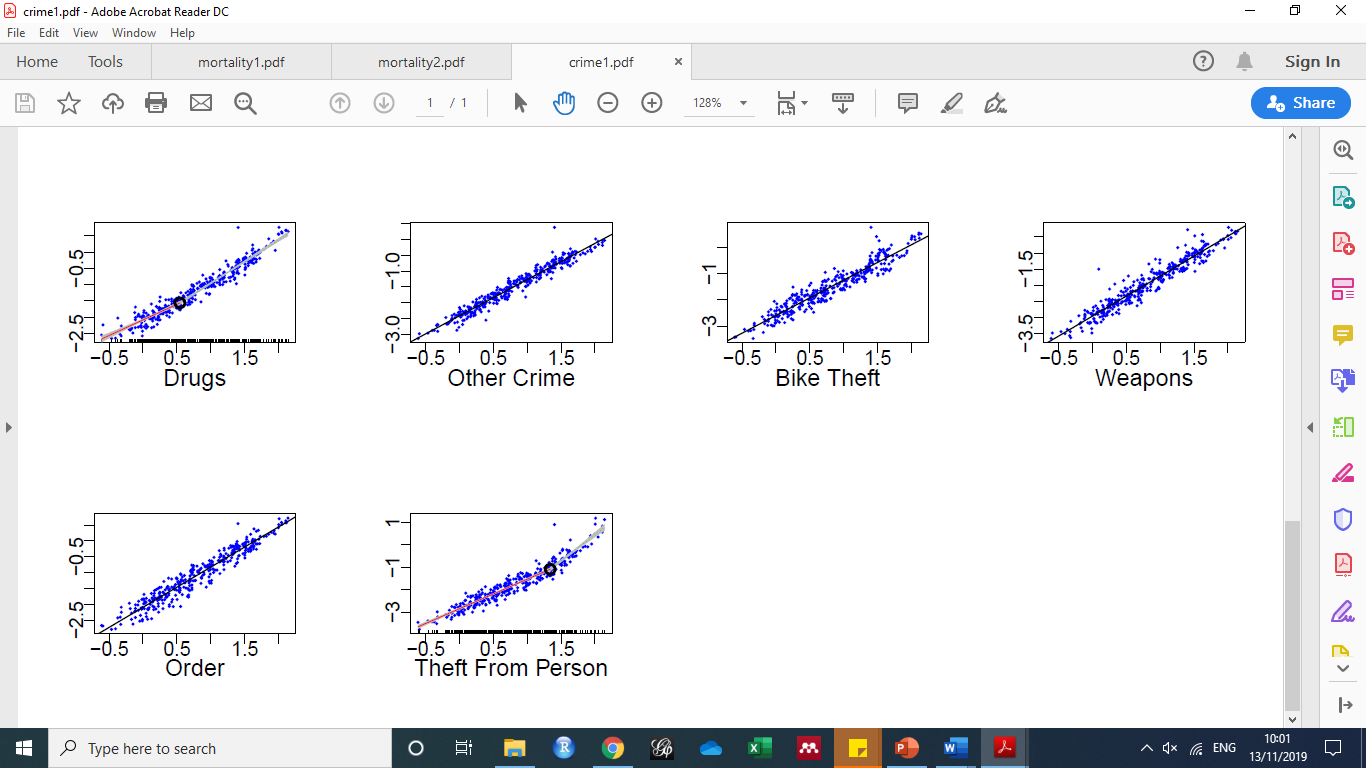 |
|  | Log(Population Density) |

**Fig S1. Density scaling behaviour of different crime types in England and Wales.** The blue dots are the empirical values. The black circle is the identified critical density $\log d^{*}$ for metrics where a double power-law is a better fit (see also S1 Table). Otherwise a black line represents a model with a single power-law. Below the black circle are the low-density data ($\log d<\log d^{*}$) and above are high density data. Gray shaded regions represent 95% CI.

| Log(Indicator Density) | 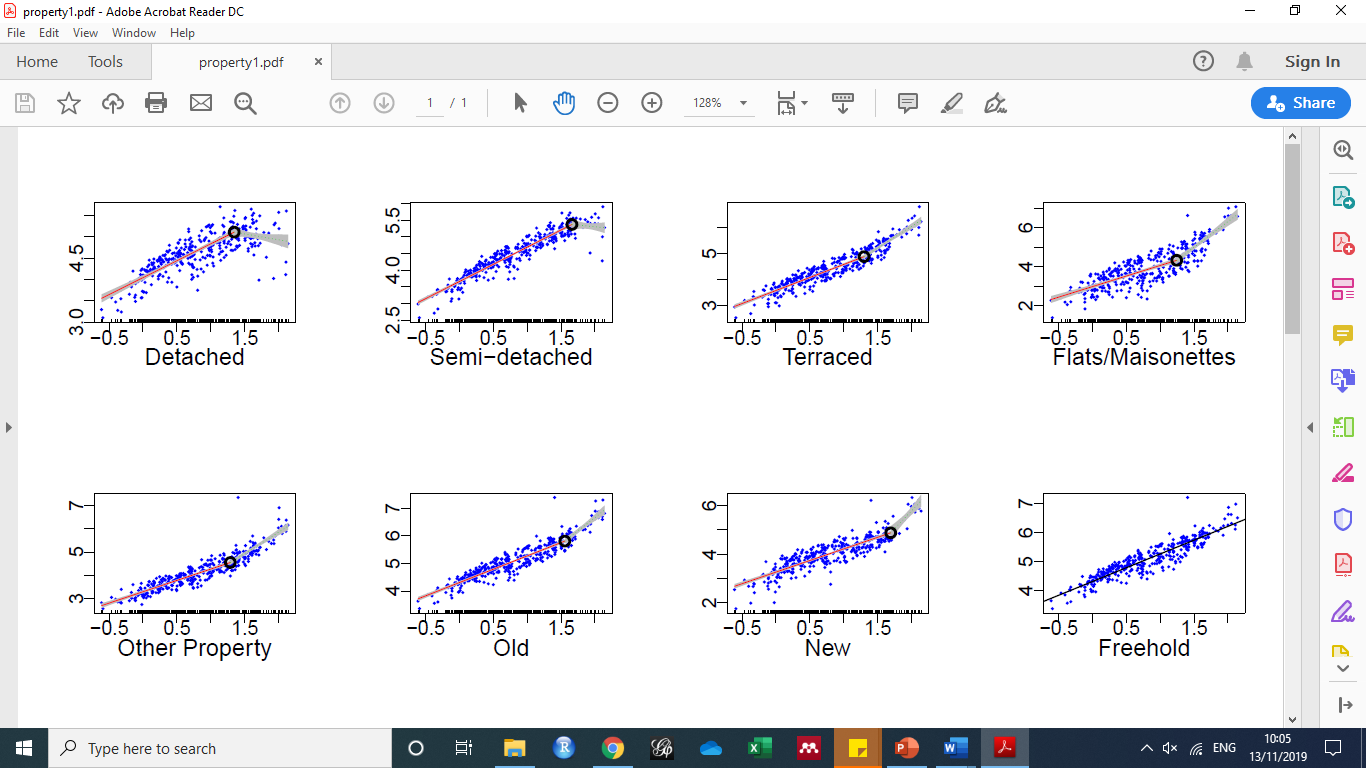 |
| --- | --- |
|  | 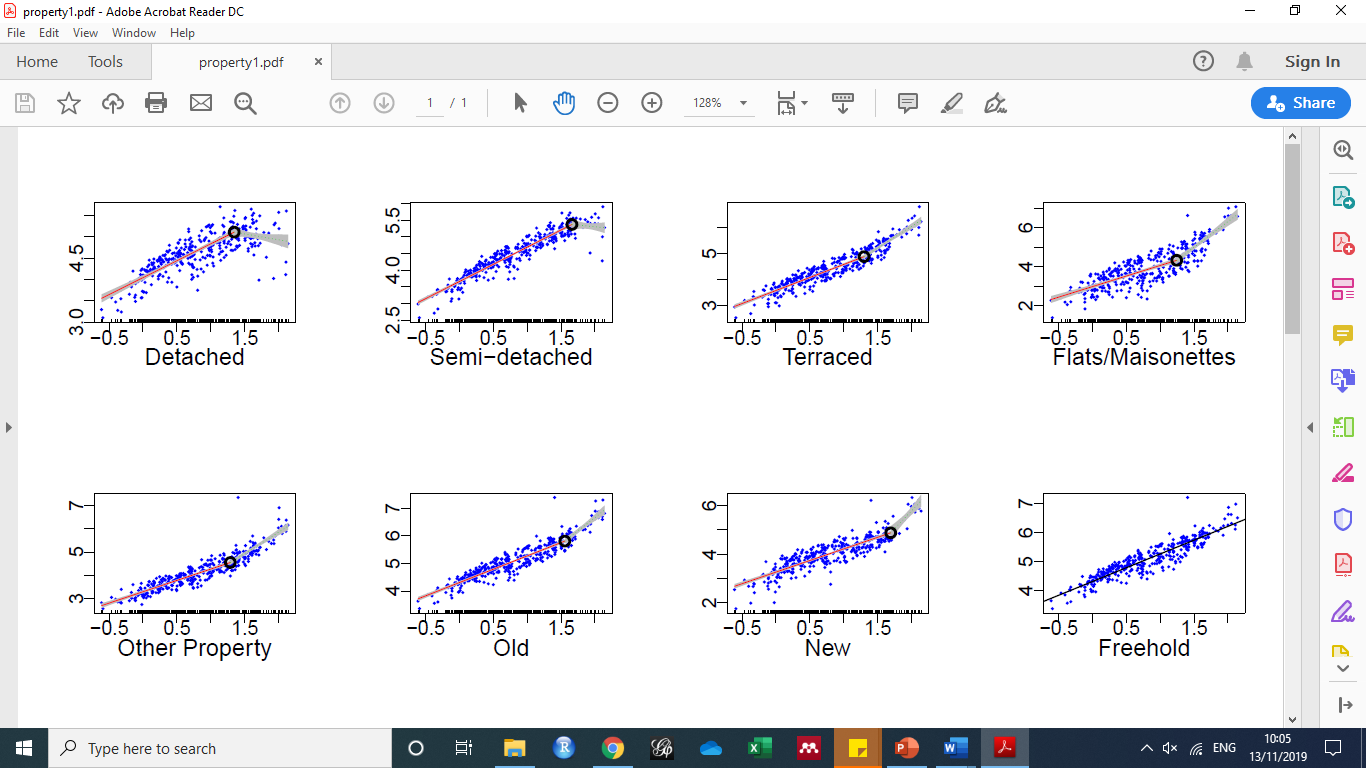 |
|  | 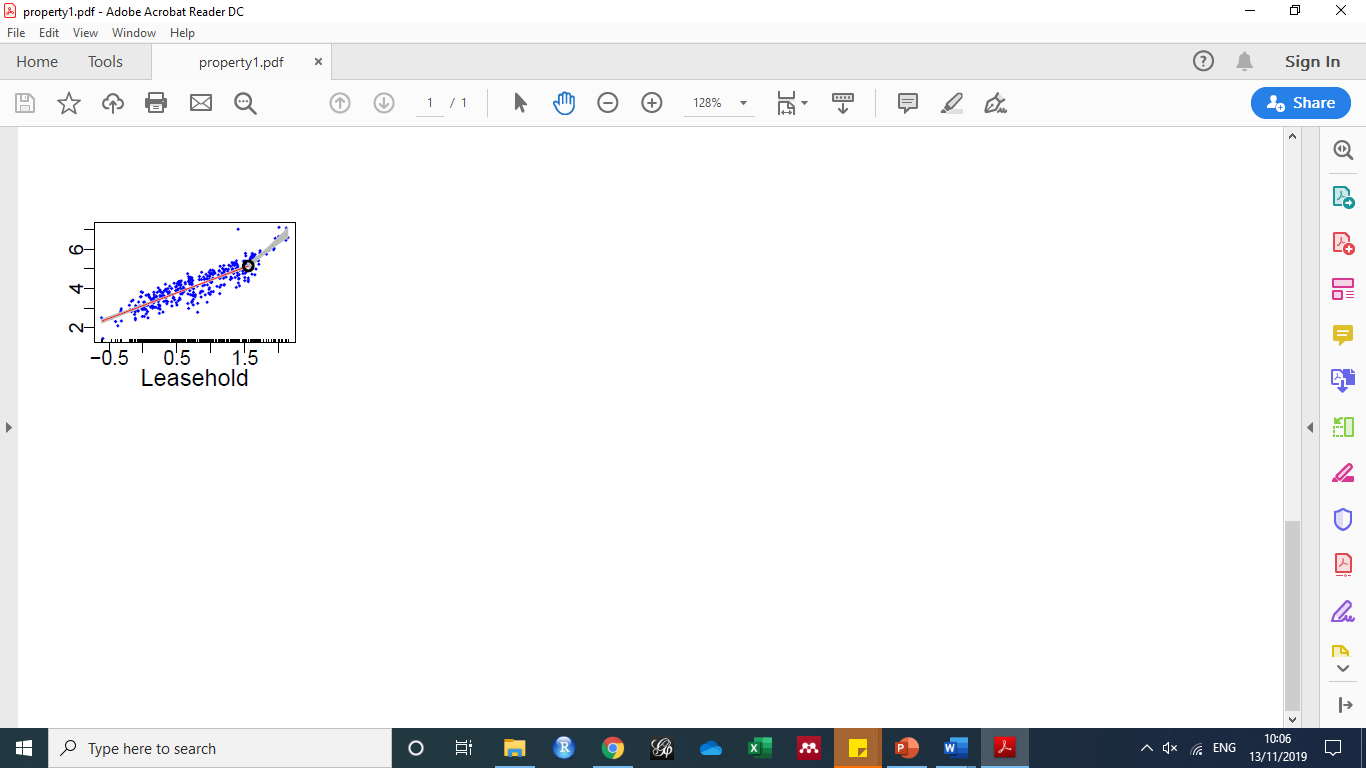 |
|  | Log(Population Density) |

**Fig S2. Density scaling behavior of different property types in England and Wales.** The blue dots are the empirical values. The black circle is the identified critical density $\log d^{*}$ for metrics where a double power-law is a better fit (see also S1 Table). Otherwise a black line represents a model with a single power-law. Below the black circle are the low-density data ($\log d<\log d^{*}$). Gray shaded regions represent 95% CI.

| Log(Indicator Density) | 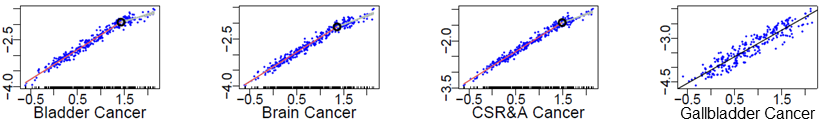 |
| --- | --- |
|  | 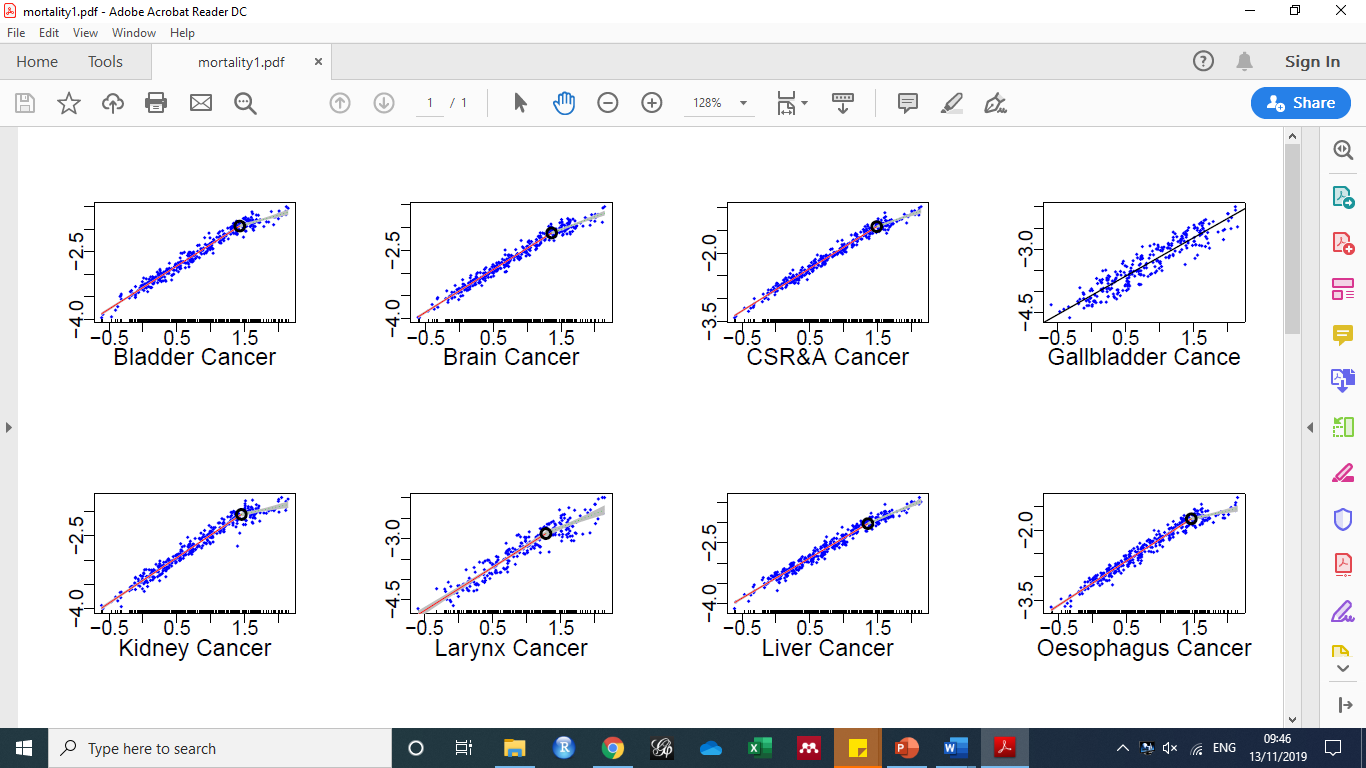 |
|  | 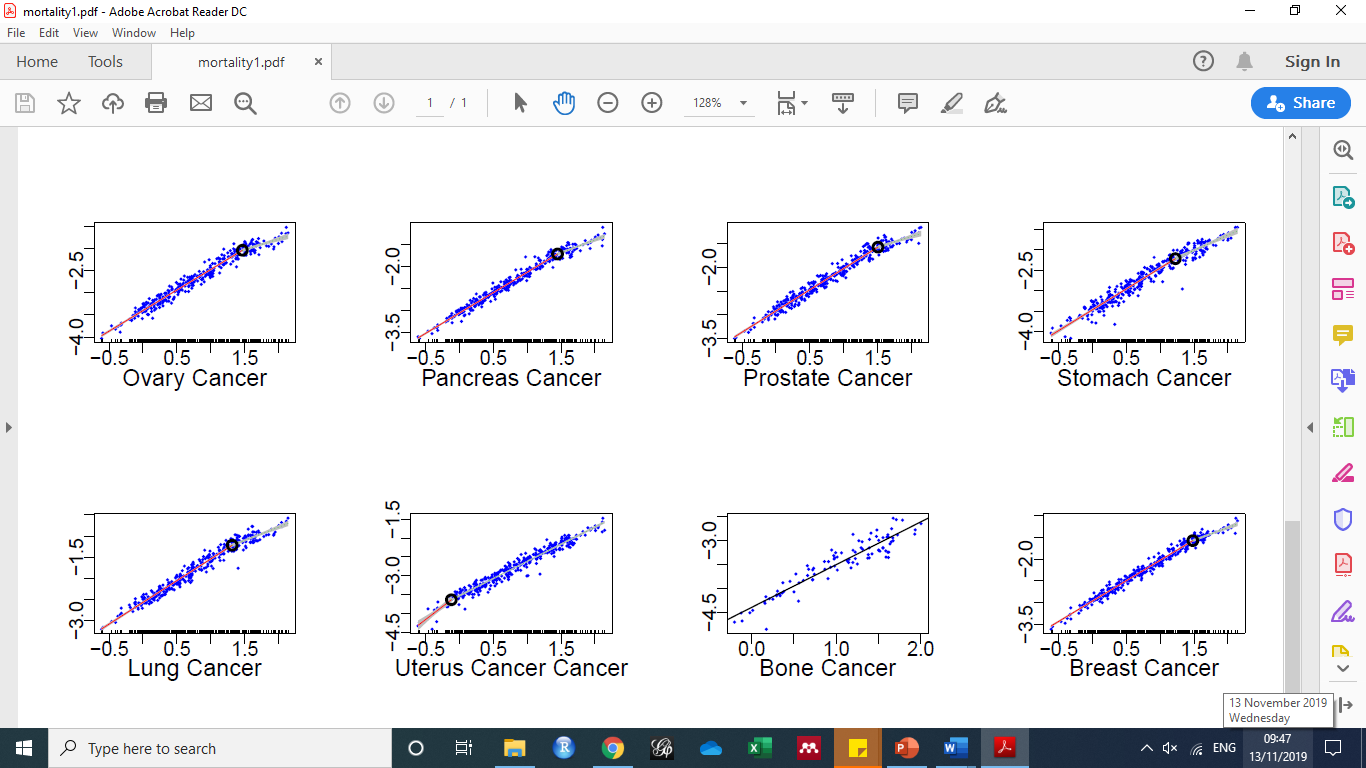 |
|  | 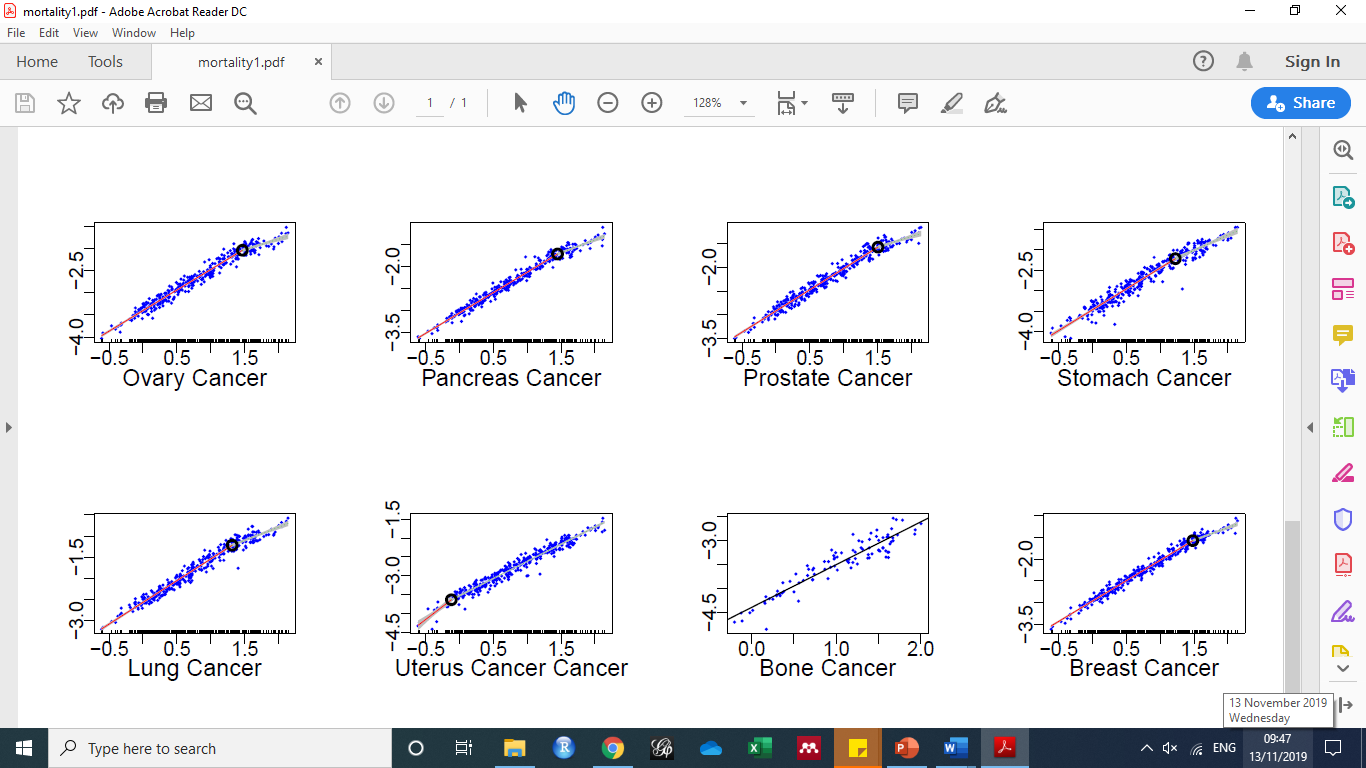 |
|  | 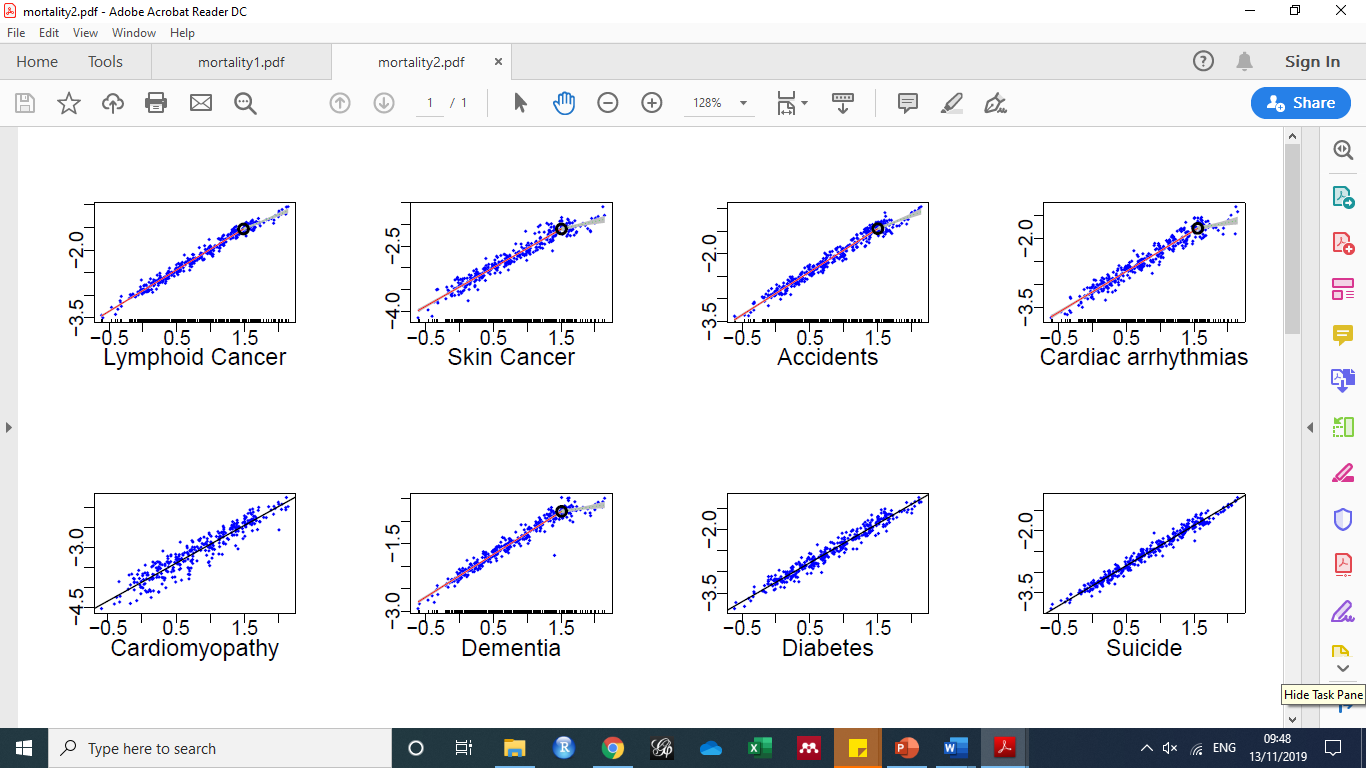 |
|  | 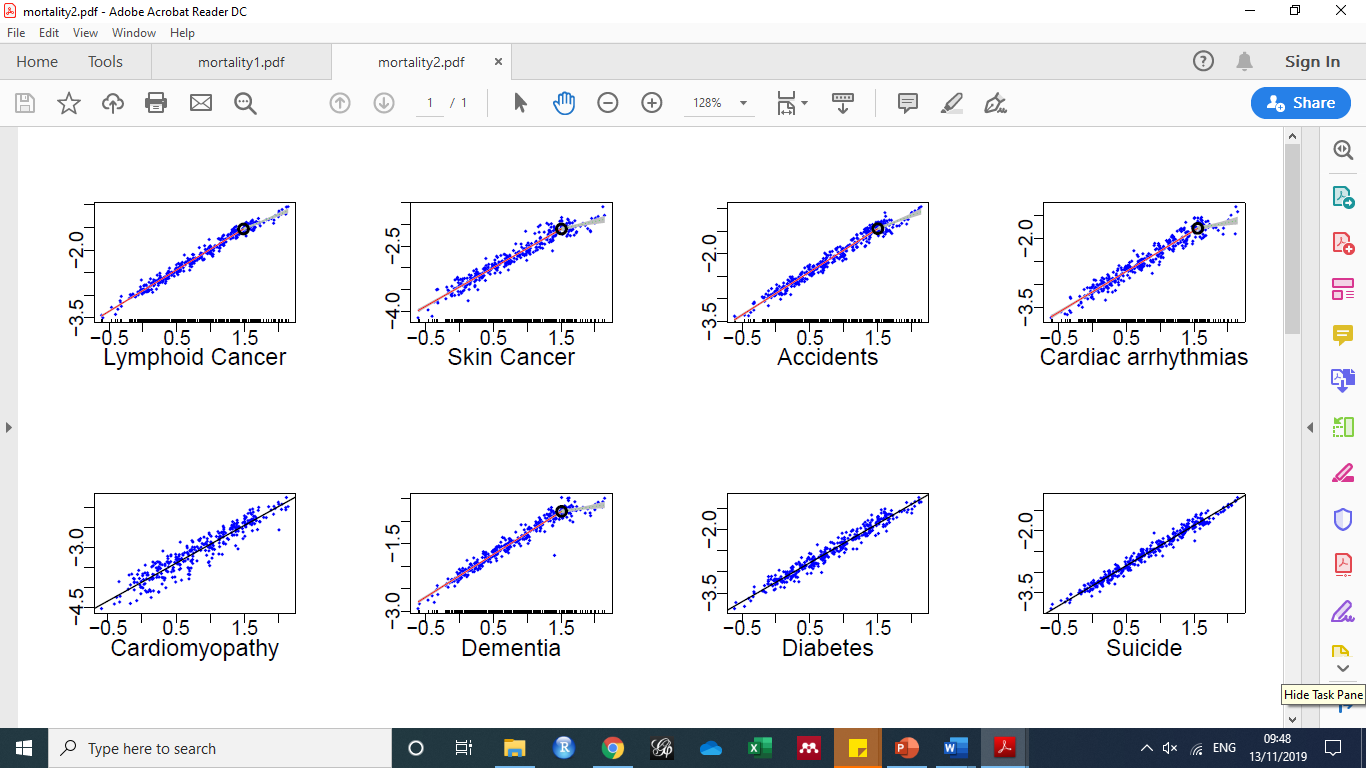 |
|  | 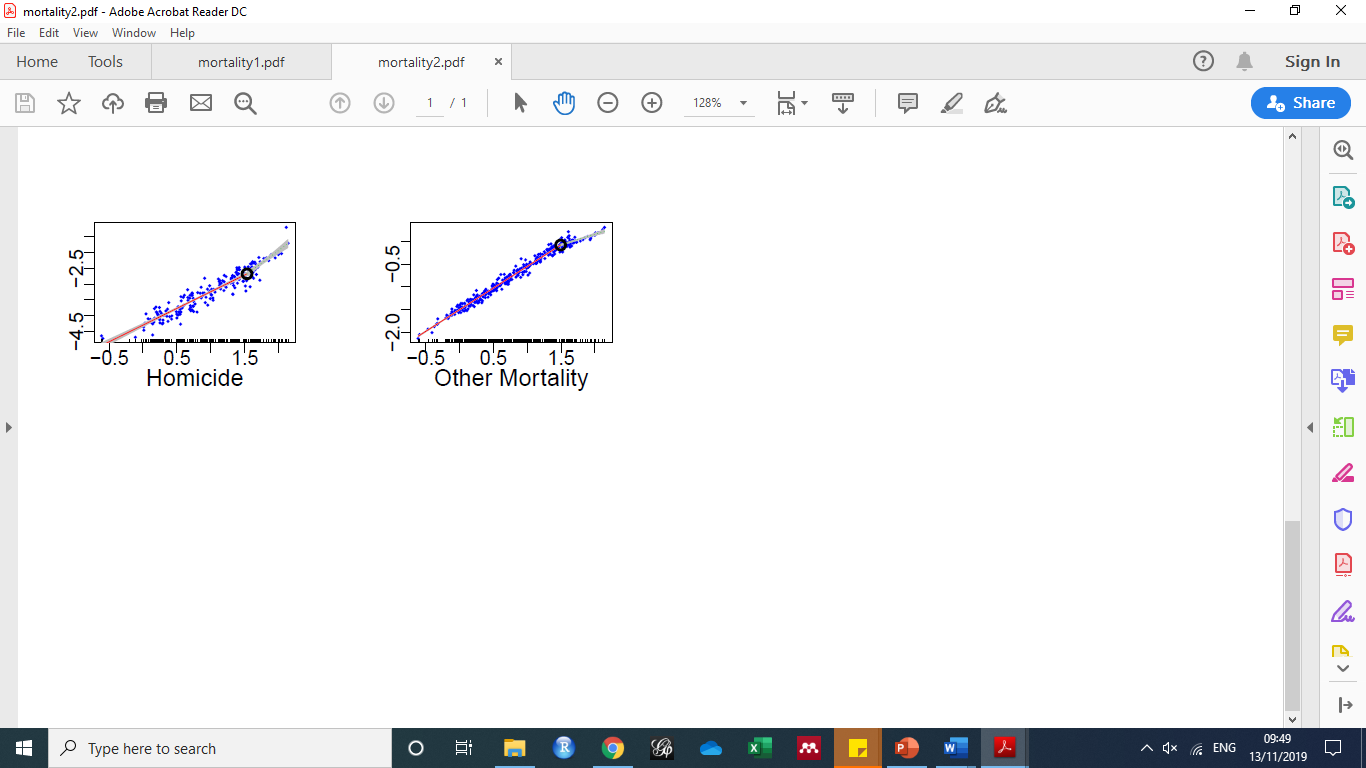 |
|  | Log(Population Density) |

**Fig S3. Density scaling behaviour of different mortality types in England and Wales.** The blue dots are the empirical values. The black circle is the identified critical density $\log d^{*}$ for metrics where a double power-law is a better fit (see also S1 Table). Otherwise a black line represents a model with a single power-law. Below the blue circle are the low-density data ($\log d<\log d^{*}$) and above are high density data. Gray shaded regions represent 95% CI.

| Log(Indicator Density) | 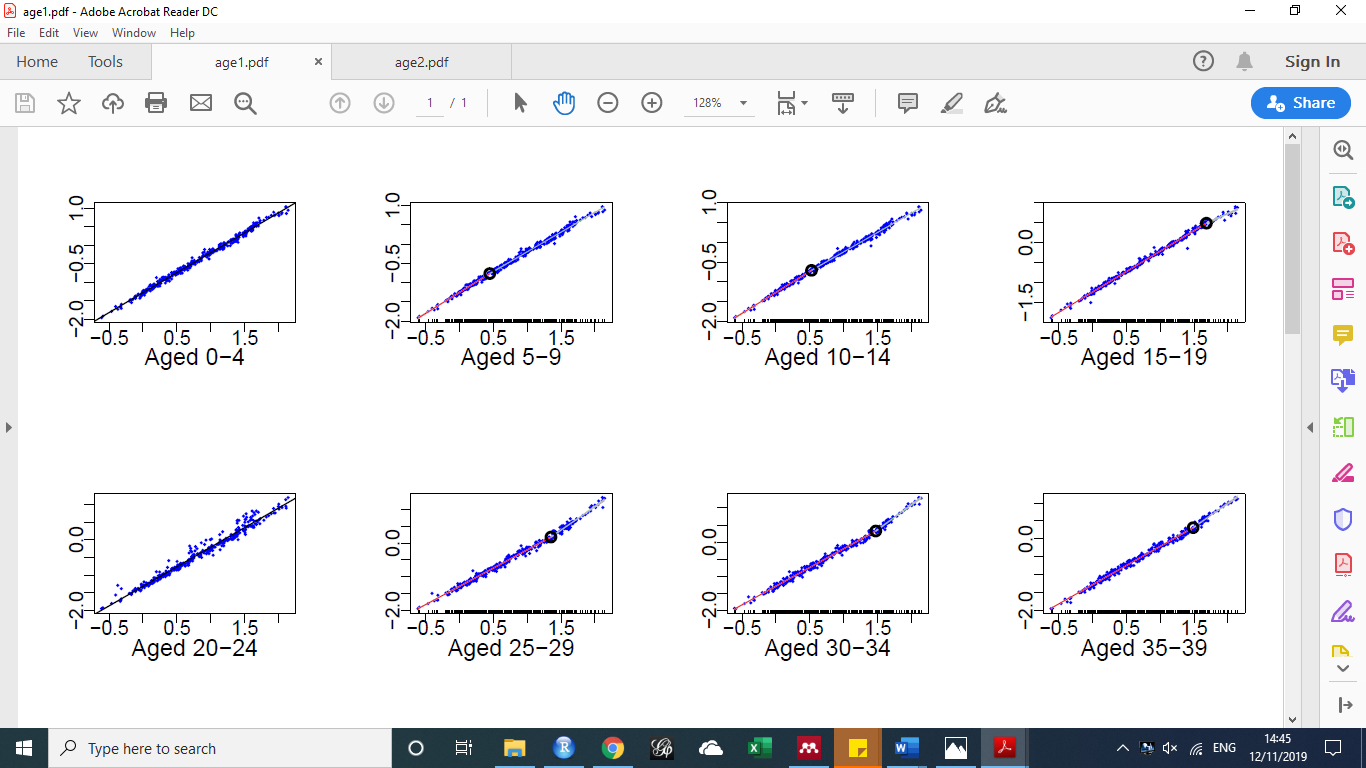 |
| --- | --- |
|  | 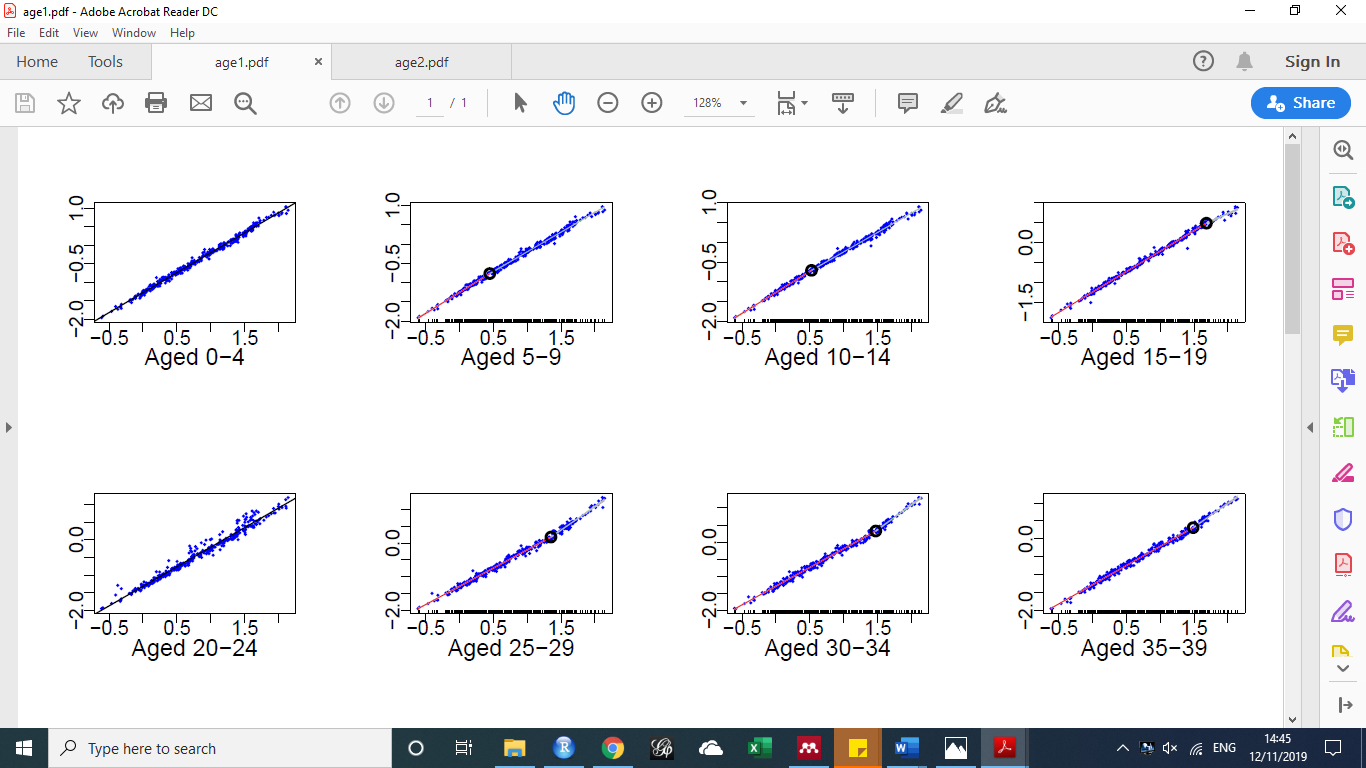 |
|  | 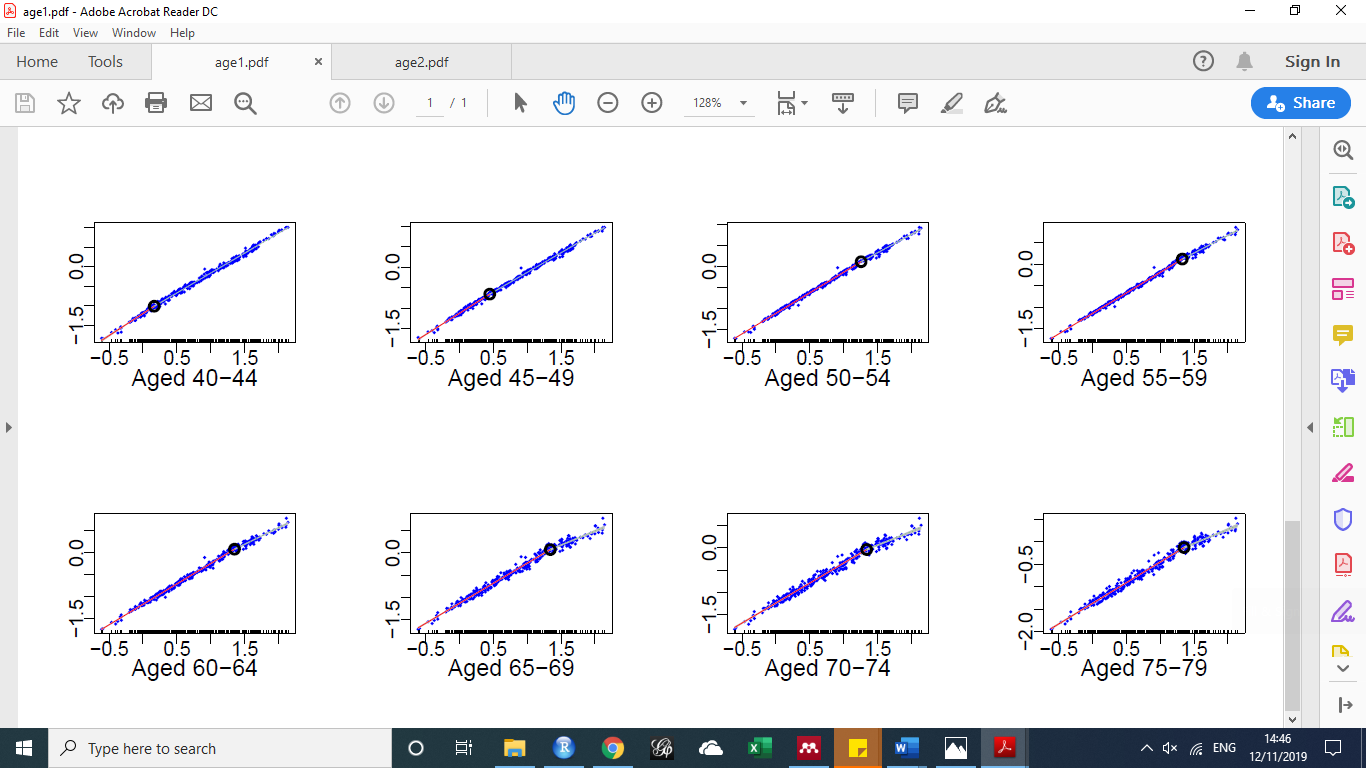 |
|  | 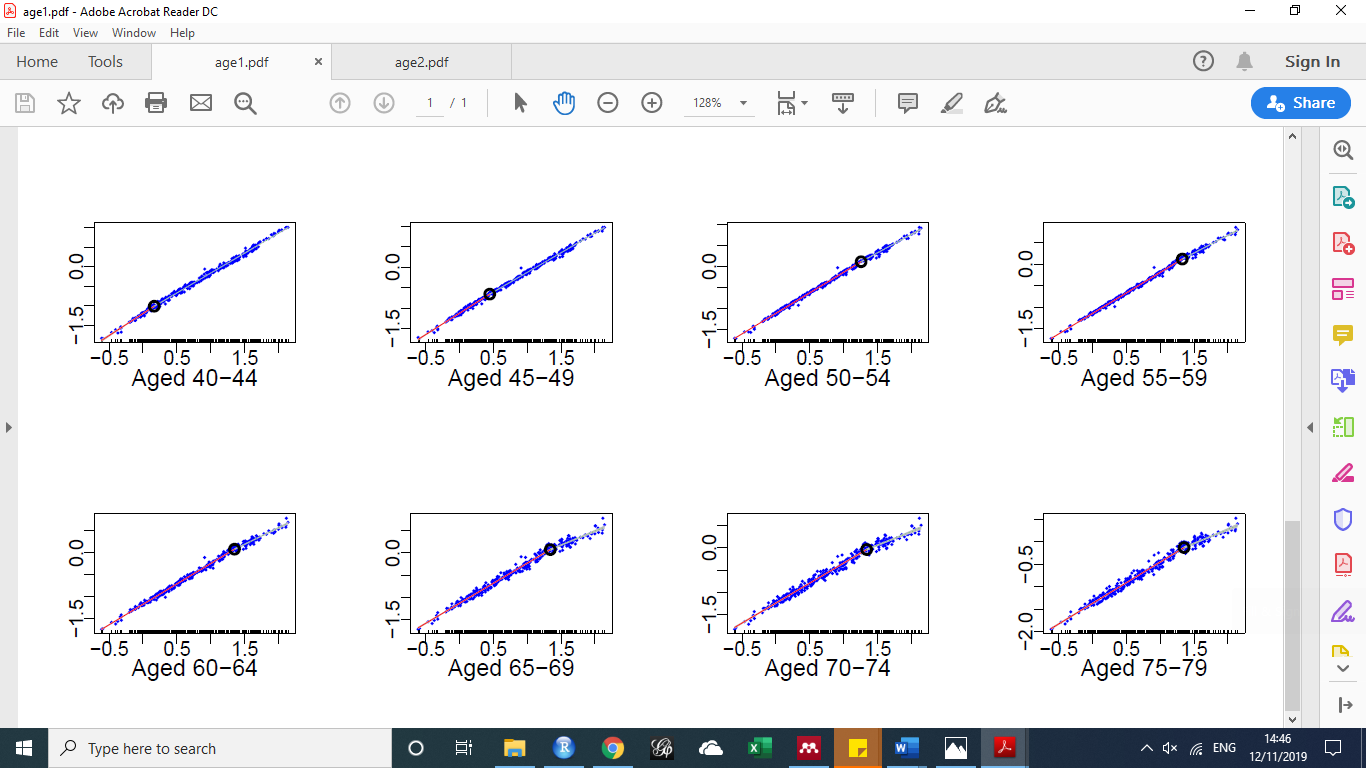 |
|  | 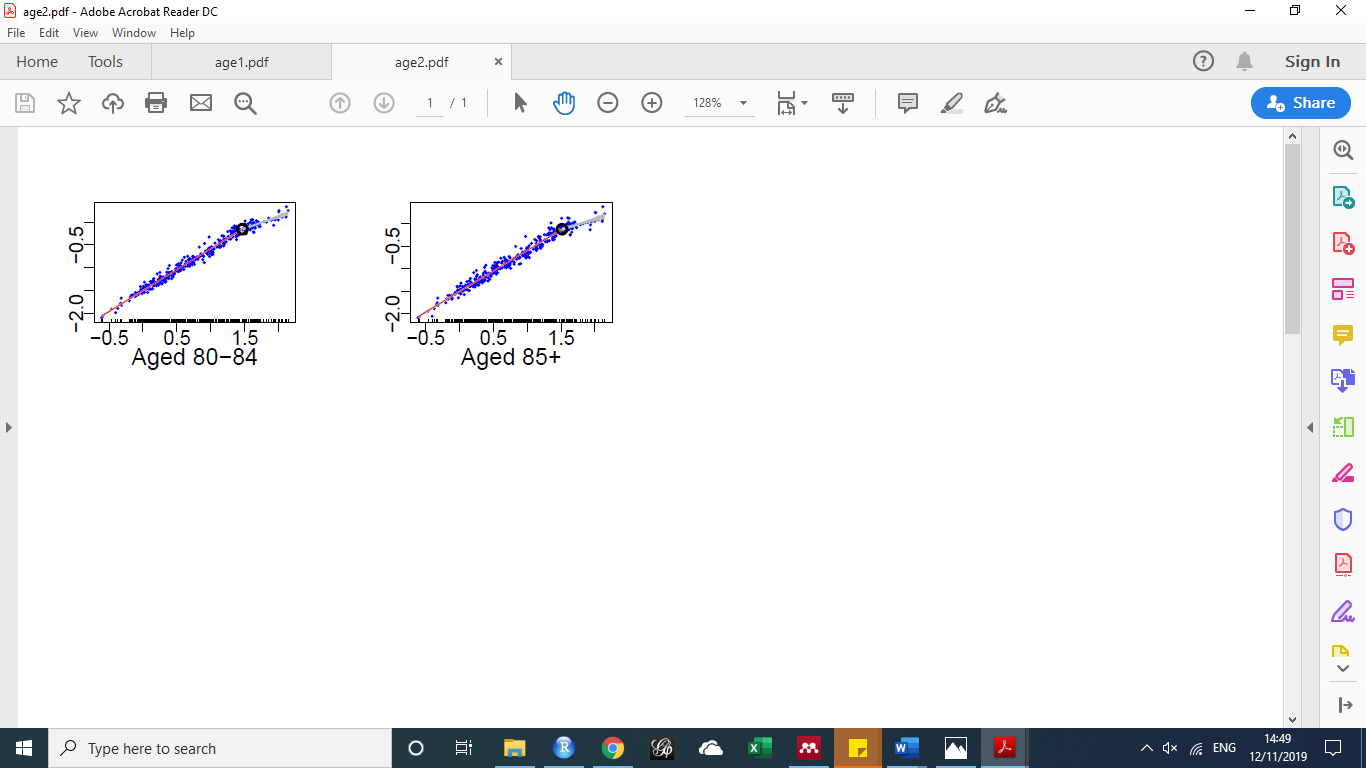 |
|  | Log(Population Density) |

**Fig S4. Density scaling behavior of different age groups in England and Wales.** The blue dots are the empirical values. The black circle is the identified critical density $\log d^{*}$ for metrics where a double power-law is a better fit (see also S1 Table). Otherwise a black line represents a model with a single power-law. Below the black circle are the low-density data ($\log d<\log d^{*}$) and above are high density data. Gray shaded regions represent 95% CI.

**Table S1. AIC AND BIC values for single and double power-law models.**

|  | Single Power-law | | Double Power-law | |
| --- | --- | --- | --- | --- |
|  | AIC | BIC | AIC | BIC |
| Crime Type |  |  |  |  |
| Anti-social behaviour ** | -215.926 | -204.369 * | -220.209 * | -200.948 |
| Burglary ** | -400.846 | -389.289 * | -401.866 * | -382.605 |
| Robbery | 26.13845 | 37.68642 | -2.9307 * | 16.31592 * |
| Vehicle Crime ** | -302.387 | -290.83 * | -307.813 * | -288.567 |
| Violent Crime ** | -412.946 * | -401.39 * | -410.406 | -391.145 |
| Shoplifting | -128.131 | -116.575 | -151.317 * | -132.056 * |
| CD and A | -427.192 | -415.636 | -474.54 * | -455.279 * |
| Other Theft | -394.604 | -383.047 | -423.971 * | -404.71 * |
| Drugs | -175.36 | -163.803 | -203.256 * | -183.995 * |
| Other Crime ** | -295.359 | -283.811 * | -302.175 * | -282.928 |
| Bike Theft ** | 82.37708 | 93.93369 * | 80.32013 * | 99.57114 |
| Weapons ** | -69.1468 * | -57.5902 * | -66.2228 | -46.9617 |
| Order ** | -65.6769 * | -54.129 * | -64.3477 | -45.1011 |
| Theft From Person | 83.09193 | 94.63991 | 15.5239 * | 34.77052 * |
| Property Type |  |  |  |  |
| Detached | 160.7479 | 172.2959 | 113.9497 * | 133.1963 * |
| Semi-detached | -45.0361 | -33.4795 | -73.4769 * | -54.2159 * |
| Terraced | 28.42889 | 39.9855 | -5.11625 * | 14.14476 * |
| Flats/Maisonettes | 551.0343 | 562.5909 | 503.118 * | 522.379 * |
| Other Property | 145.4305 | 156.9871 | 103.7093 * | 122.9703 * |
| Old | 30.99874 | 42.55535 | -0.58146 * | 18.67955 * |
| New | 238.3159 | 249.8725 | 197.9525 * | 217.2135 * |
| Freehold ** | 18.84382 | 30.40043 * | 13.71841 * | 32.97942 |
| Leasehold | 391.0368 | 402.5934 | 357.9221 * | 377.1831 * |
| Mortality Types |  |  |  |  |
| Other Mortality |  |  | -861.1671 | -841.9061 |
| Accidents | -589.094 | -577.555 | -623.775 * | -604.543 * |
| Bladder Cancer | -578.93 | -567.391 | -683.142 * | -663.91 * |
| Brain Cancer | -647.257 | -635.717 | -723.434 * | -704.202 * |
| CSR&A Cancer | -750.93 | -739.391 | -832.815 * | -813.583 * |
| Gallbladder Cancer ** | -67.2978 | -56.57 * | -68.9083 * | -51.0286 |
| Kidney Cancer | -478.835 | -467.304 | -586.334 * | -567.116 * |
| Larynx Cancer | -94.318 | -83.9907 | -107.358 * | -90.1459 * |
| Liver Cancer | -601.064 | -589.516 | -634.615 * | -615.368 * |
| Oesophagus Cancer | -525.709 | -514.17 | -642.599 * | -623.367 * |
| Ovary Cancer | -568.79 | -557.25 | -630.325 * | -611.093 * |
| Pancreas Cancer | -710.711 | -699.171 | -767.232 * | -748 * |
| Prostate Cancer | -633.503 | -621.964 | -694.028 * | -674.796 * |
| Stomach Cancer | -369.671 | -358.132 | -389.694 * | -370.462 * |
| Lung Cancer | -514.186 | -502.638 | -578.028 * | -558.781 * |
| Uterus Cancer | -509.094 | -497.572 | -518.602 * | -499.399 * |
| Bone Cancer ** | -48.427 | -40.7029 | -54.0867 * | -41.2132 * |
| Breast Cancer | -751.173 | -739.625 | -813.289 * | -794.042 * |
| Lymphoid Cancer | -775.184 | -763.636 | -820.265 * | -801.019 * |
| Skin Cancer | -432.399 | -420.859 | -482.357 * | -463.125 * |
| Cardiac arrhythmias | -428.249 | -416.71 | -479.676 * | -460.444 * |
| Cardiomyopathy ** | -114.03 * | -102.697 * | -112.306 | -93.4178 |
| Dementia | -458.862 | -447.305 | -566.233 * | -546.972 * |
| Diabetes ** | -505.221 * | -493.681 * | -504.752 | -485.52 |
| Homicide | -48.6574 | -38.7775 | -57.9741 * | -41.5076 * |
| Suicide ** | -630.617 | -619.077 * | -631.061 * | -611.829 |
| Age Type |  |  |  |  |
| Aged 0-4 ** | -1038.28 | -1026.73 * | -1040.92 * | -1021.66 |
| Aged 5-9 | -1142.27 | -1130.71 | -1153.41 * | -1134.15 * |
| Aged 10-14 | -1186.53 | -1174.97 | -1206.91 * | -1187.65 * |
| Aged 15-19 | -1131.15 | -1119.59 | -1172.68 * | -1153.42 * |
| Aged 20-24 ** | -557.687 | -546.131 * | -562.883 * | -543.622 |
| Aged 25-29 | -844.804 | -833.247 | -937.294 * | -918.033 * |
| Aged 30-34 | -890.689 | -879.132 | -992.651 * | -973.39 * |
| Aged 35-39 | -990.891 | -979.334 | -1045.62 * | -1026.36 * |
| Aged 40-44 | -1201.11 | -1189.56 | -1217.16 * | -1197.9 * |
| Aged 45-49 | -1343.34 | -1331.79 | -1381.15 * | -1361.89 * |
| Aged 50-54 | -1361.57 | -1350.01 | -1410.26 * | -1391 * |
| Aged 55-59 | -1315.45 | -1303.9 | -1387.65 * | -1368.39 * |
| Aged 60-64 | -1147.88 | -1136.32 | -1221.7 * | -1202.44 * |
| Aged 65-69 | -892.011 | -880.455 | -977.202 * | -957.941 * |
| Aged 70 74 | -816.243 | -804.686 | -892 * | -872.739 * |
| Aged 75 79 | -818.535 | -806.978 | -895.91 * | -876.649 * |
| Aged 80-84 | -754.224 | -742.668 | -836.673 * | -817.412 * |
| Aged 85+ | -638.906 | -627.349 | -700.526 * | -681.265 * |

* indicates better model based on the AIC/BIC score

** indicates that the Davies test did not detect a bend in the model therefore single power-law model is preferred.

**Table S2. A list of all low and high exponents, critical densities (if applicable) and intercepts.**

| Crime Type | Log(Y_0_) | β  (Low Density) | log(d*)  (Critical Density Threshold) | β*  (High Density) |
| --- | --- | --- | --- | --- |
| ASB | -0.94 [CI: -0.99 -0.92] | 1.13 [CI: 1.10 1.16] | - | - |
| Bike Theft | -2.60 [CI: -2.63 -2.54] | 1.35 [CI: 1.30 1.40] | - | - |
| Burglary | -1.63 [CI: -1.67 -1.62] | 1.16 [CI: 1.14 1.18] | - | - |
| CD & A | -1.48 [CI: -1.50 -1.46] | 1.16 [CI: 1.13 1.19] | 1.41 [CI: 1.29 1.53] | 0.71 [CI: 0.56 0.85] |
| Drugs | -2.09 [CI: -2.12 -2.05] | 0.96 [CI: 0.84 1.08] | 0.55 [CI: 0.35 0.74] | 1.33 [CI: 1.27 1.39] |
| Order | -2.09 [CI: -2.14 -2.06] | 1.27 [CI: 1.23 1.30] | - | - |
| Other Crime | -2.41 [CI: -2.46 -2.40] | 1.14 [CI: 1.11 1.16] | - | - |
| Other Theft | -1.54 [CI: -1.56 -1.52] | 1.14 [CI: 1.11 1.16] | 1.77 [CI: 1.61 1.94] | 2.04 [CI: 1.43 2.64] |
| Robbery | -3.15 [CI: -3.19 -3.10] | 1.51 [CI: 1.45 1.58] | 1.38 [CI: 1.23 1.53] | 2.20 [CI: 1.93 2.46] |
| Shoplifting | -1.82 [CI: -1.85 -1.78] | 1.28 [CI: 1.24 1.33] | 1.45 [CI: 1.28 1.61] | 0.73 [CI: 0.48 0.98] |
| Theft from Person | -2.85 [CI: -2.90 -2.81] | 1.31 [CI: 1.25 1.38] | 1.34 [CI: 1.24 1.44] | 2.32 [CI: 2.07 2.56] |
| Vehicle Crime | -1.80 [CI: -1.84 -1.79] | 1.27 [CI: 1.24 1.29] | - | - |
| Violent Crimes | -1.25 [CI: -1.27 -1.23] | 1.17 [CI: 1.15 1.20] | - | - |
| Weapons | -2.96 [CI: -3.01 -2.93] | 1.24 [CI: 1.21 1.28] | - | - |
| Property Type |  |  |  |  |
| Detached | 4.04 [CI: 3.98 4.10] | 0.80 [CI: 0.72 0.87] | 1.35 [CI: 1.24 1.46] | -0.27 [CI: -0.56 0.01] |
| Flats | 2.99 [CI: 2.89 3.08] | 1.08 [CI: 0.93 1.23] | 1.24 [CI: 1.11 1.38] | 2.62 [CI: 2.18 3.05] |
| Freehold | 4.30 [CI: 4.28 4.37] | 0.95 [CI: 0.91 0.99] | - | - |
| Leasehold | 3.12 [CI: 3.06 3.18] | 1.31 [CI: 1.22 1.39] | 1.56 [CI: 1.43 1.68] | 2.94 [CI: 2.28 3.61] |
| New | 3.26 [CI: 3.20 3.32] | 0.95 [CI: 0.89 1.01] | 1.69 [CI: 1.55 1.84] | 2.92 [CI: 1.94 3.90] |
| Old | 4.31 [CI: 4.26 4.35] | 0.97 [CI: 0.92 1.02] | 1.56 [CI: 1.43 1.68] | 1.92 [CI: 1.53 2.32] |
| Semi-detached | 3.66 [CI: 3.62 3.70] | 1.03 [CI: 0.99 1.02] | 1.64 [CI: 1.52 1.77] | -0.14 [CI: -0.63 0.34] |
| Terraced | 3.58 [CI: 3.53 3.62] | 1.01 [CI: 0.94 1.08] | 1.30 [CI: 1.15 1.45] | 1.67 [CI: 1.45 1.89] |
| Other Property | 3.27 [CI: 3.22 3.33] | 0.98 [CI: 0.90 1.06] | 1.29 [CI: 1.15 1.43] | 1.83 [CI: 1.57 2.08] |
| Mortality Types |  |  |  |  |
| Accidents | -2.88 [CI: -2.90 -2.86] | 0.96 [CI: 0.93 0.98] | 1.51 [CI: 1.39 1.62] | 0.59 [CI: 0.45 0.72] |
| Bladder Cancer | -3.30 [CI: -3.31 -3.28] | 0.95 [CI: 0.93 0.98] | 1.43 [CI: 1.35 1.51] | 0.43 [CI: 0.31 0.54] |
| Brain Cancer | -3.39 [CI: -3.41 -3.37] | 0.94 [CI: 0.91 0.96] | 1.36 [CI: 1.27 1.46] | 0.56 [CI: 0.47 0.65] |
| CSR and A Cancer | -2.80 [CI: -2.82 -2.79] | 0.93 [CI: 0.91 0.94] | 1.49 [CI: 1.41 1.57] | 0.52 [CI: 0.42 0.61] |
| Gallbladder Cancer | -4.10 [CI: -4.16 -4.08] | 0.91 [CI: 0.87 0.96] | - | - |
| Kidney Cancer | -3.42 [CI: -3.44 -3.40] | 0.93 [CI: 0.91 0.96] | 1.45 [CI: 1.38 1.53] | 0.30 [CI: 0.16 0.43] |
| Larynx Cancer | -4.23 [CI: -4.29 -4.18] | 1.06 [CI: 0.99 1.13] | 1.28 [CI: 1.04 1.52] | 0.67 [CI: 0.48 0.86] |
| Liver Cancer | -3.37 [CI: -3.39 -3.35] | 0.99 [CI: 0.97 1.02] | 1.36 [CI: 1.21 1.50] | 0.71 [CI: 0.61 0.81] |
| Oesophagus Cancer | -3.13 [CI: -3.16 -3.11] | 0.94 [CI: 0.92 0.97] | 1.46[CI: 1.39 1.53] | 0.32 [CI: 0.19 0.44] |
| Ovary Cancer | -3.40 [CI: -3.42 -3.38] | 0.93 [CI: 0.90 0.95] | 1.47 [CI: 1.37 1.56] | 0.47[CI: 0.34 0.59] |
| Pancreas Cancer | -3.06 [CI: -3.07 -3.04] | 0.93 [CI: 0.91 0.95] | 1.45 [CI: 1.34 1.55] | 0.58 [CI: 0.48 0.68] |
| Prostate Cancer | -2.92 [CI: -2.93 -3.04] | 0.89 [CI: 0.87 0.91] | 1.51 [CI: 1.41 1.60] | 0.45 [CI: 0.33 0.57] |
| Stomach Cancer | -3.46 [CI: -3.49 -3.43] | 1.02 [CI: 0.98 1.06] | 1.23 [CI: 1.01 1.44] | 0.74 [CI: 0.62 0.86] |
| Lung Cancer | -2.57 [CI: -2.59 -2.55] | 1.03 [CI: 1.00 1.05] | 1.32 [CI: 1.21 1.43] | 0.62 [CI: 0.52 0.72] |
| Uterus Cancer | -3.47 [CI: -3.61 -3.34] | 1.38 [CI: 1.00 1.76] | -0.12[CI: -0.33 0.09] | 0.91 [CI: 0.89 0.93] |
| Bone Cancer | -4.39 [CI: -4.57 -4.41] | 0.89 [CI: 0.83 0.96] | - | - |
| Breast Cancer | -2.96 [CI: -2.98 -2.95] | 0.94 [CI: 0.92 0.96] | 1.48 [CI: 1.39 1.57] | 0.58 [CI: 0.48 0.68] |
| Lymphoid Cancer | -2.89 [CI: -2.91 -2.88] | 0.92 [CI: 0.90 0.94] | 1.48 [CI: 1.37 1.59] | 0.61 [CI: 0.51 0.71] |
| Skin Cancer | -3.44 [CI: -3.47 -3.42] | 0.89 [CI: 0.86 0.92] | 1.51 [CI: 1.41 1.61] | 0.35 [CI: 0.18 0.51] |
| Cardiac arrhythmias | -3.16 [CI: -3.19 -3.14] | 0.89 [CI: 0.87 0.92] | 1.55 [CI: 1.46 1.65] | 0.29 [CI: 0.09 0.48] |
| Cardiomyopathy | -3.87 [CI: -3.92 -3.84] | 0.95 [CI: 0.91 0.99] | - | - |
| Dementia | -2.22 [CI: -2.24 -2.20] | 0.95 [CI: 0.93 0.98] | 1.49 [CI: 1.43 1.56] | 0.25 [CI: 0.10 0.39] |
| Diabetes | -3.25 [CI: -3.27 -3.23] | 0.92 [CI: 0.90 0.94] | - | - |
| Suicide | -3.33 [CI: -3.35 -3.32] | 0.97[CI: 0.96 0.99] | - | - |
| Homicide | -4.29 [CI: -4.35 -4.23] | 1.05 [CI: 0.99 1.12] | 1.53 [CI: 1.31 1.75] | 1.57[CI: 1.24 1.90] |
| Other Mortality | -1.50 [CI: -1.52 -1.48] | 0.95 [CI: 0.93 0.97] | 1.49 [CI: 1.43 1.56] | 0.47 [CI: 0.37 0.56] |
| Age Categories |  |  |  |  |
| Age 0 - 4 | -1.28 [CI: -1.29 -1.27] | 1.08 [CI: 1.07 1.09] | - | - |
| Aged 5-9 | -1.26 [CI: -1.27 -1.25] | 1.09 [CI: 1.06 1.13] | 0.45 [CI: 0.18 0.72] | 1.02 [CI: 1.01 1.04] |
| Aged 10-14 | -1.26 [CI: -1.27 -1.26] | 1.06 [CI: 1.03 1.09] | 0.52 [CI: 0.30 0.75] | 0.98 [CI: 0.97 1.00] |
| Aged 15-19 | -1.24 [CI: -1.25 -1.23] | 1.02 [CI: 1.01 1.03] | 1.68 [CI: 1.54 1.82] | 0.75 [CI: 0.62 0.88] |
| Aged 20-24 | -1.31 [CI: -1.33 -1.29] | 1.11 [CI: 1.09 1.12] | - | - |
| Aged 25-29 | -1.28 [CI: -1.30 -1.27] | 1.08 [CI: 1.06 1.10] | 1.35 [CI: 1.27 1.44] | 1.39 [CI: 1.32 1.45] |
| Aged 30-34 | -1.29 [CI: -1.30 -1.28] | 1.10 [CI: 1.08 1.11] | 1.47 [CI: 1.40 1.54] | 1.46 [CI: 1.38 1.53] |
| Aged 35-39 | -1.28 [CI: -1.29 -1.27] | 1.07 [CI: 1.06 1.08] | 1.49 [CI: 1.39 1.59] | 1.32 [CI: 1.24 1.39] |
| Aged 40-44 | -1.20 [CI: -1.21 -1.19] | 1.13 [CI: 1.07 1.18] | 0.17 [CI: -0.01 0.34] | 1.02 [CI: 1.01 1.03] |
| Aged 45-49 | -1.13 [CI: -1.13 -1.12] | 1.05 [CI: 1.02 1.07] | 0.45 [CI: 0.29 0.61] | 0.96 [CI: 0.95 0.97] |
| Aged 50-54 | -1.11 [CI: -1.12 -1.10] | 0.98 [CI: 0.97 0.99] | 1.26 [CI: 1.12 1.39] | 0.88 [CI: 0.85 0.91] |
| Aged 55-59 | -1.15 [CI: 1.16 -1.14] | 0.96 [CI: 0.95 0.97] | 1.33 [CI: 1.23 1.43] | 0.82 [CI: 0.79 0.85] |
| Aged 60-64 | -1.17 [CI: -1.18 -1.16] | 0.93 [CI: 0.92 0.94] | 1.35 [CI: 1.25 1.45] | 0.75 [CI: 0.71 0.79] |
| Aged 65-69 | -1.13 [CI: -1.15 -1.12] | 0.90 [CI: 0.89 0.92] | 1.34 [CI: 1.25 1.43] | 0.63 [CI: 0.57 0.69] |
| Aged 70-74 | -1.24 [CI: -1.25 -1.22] | 0.90 [CI: 0.88 0.91] | 1.34 [CI: 1.24 1.44] | 0.61 [CI: 0.54 0.67] |
| Aged 75-79 | -1.37 [CI: -1.38 -1.35] | 0.92 [CI: 0.90 0.93] | 1.35 [CI: 1.26 1.45] | 0.62 [CI: 0.55 0.69] |
| Aged 80-84 | -1.50 [CI: -1.51 -1.49] | 0.92 [CI: 0.90 0.93] | 1.47 [CI: 1.39 1.55] | 0.52 [CI: 0.43 0.61] |
| Aged 85+ | -1.51 [CI: -1.52 -1.49] | 0.91 [CI: 0.89 0.93] | 1.51 [CI: 1.42 1.61] | 0.46 [CI: 0.33 0.59] |

| 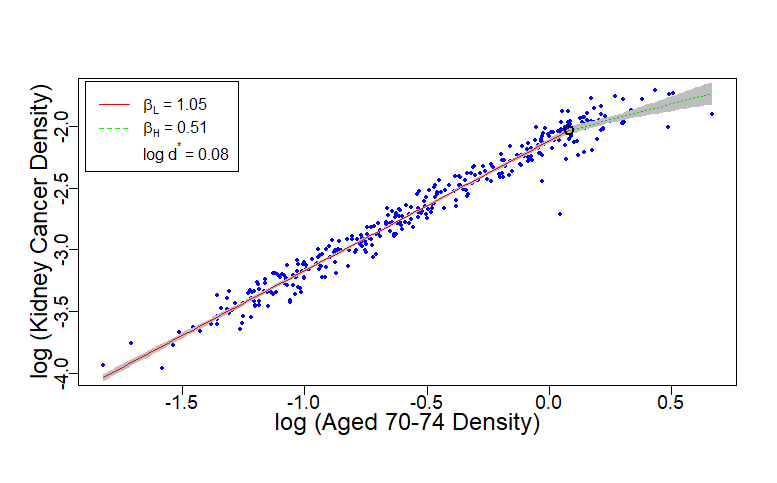 | 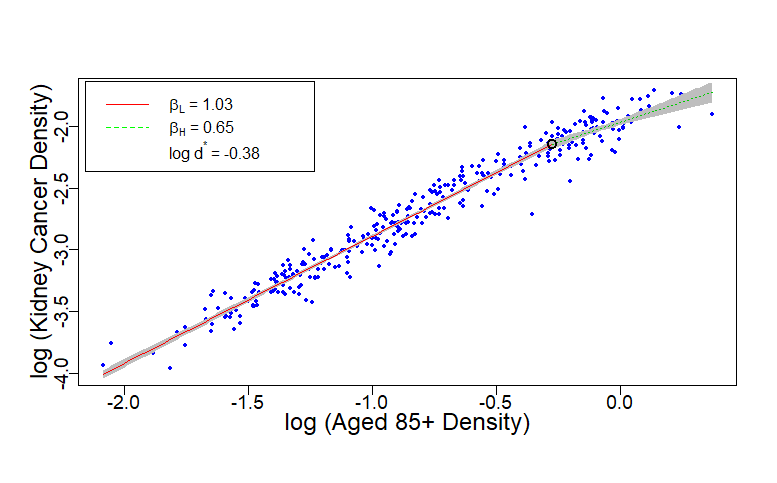 |
| --- | --- |
| (a) | (b) |
| 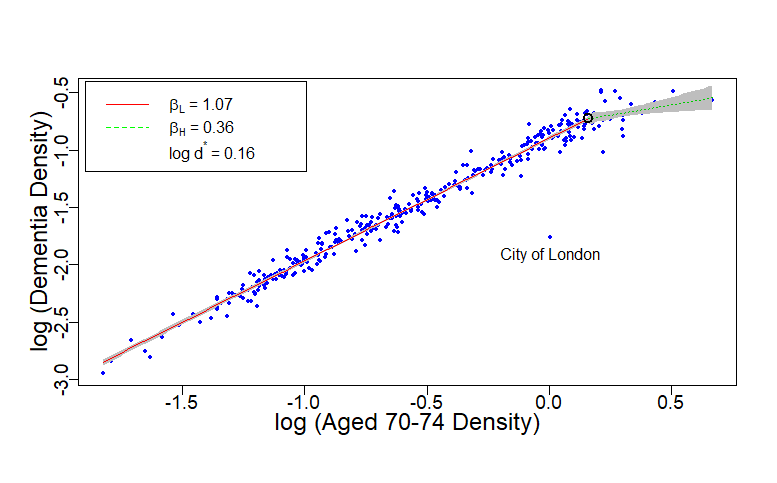 | 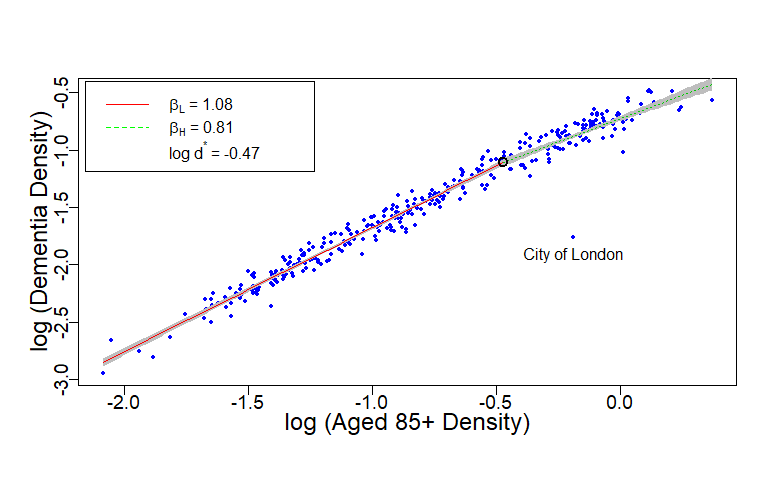 |
| (c) | (d) |

**Figure S5**: Density scaling against specific age groups. The magnitude of the exponent changes in these cases becoming less pronounced. Note: the value of the critical density is not directly comparable with those obtained from the overall population. Each age group is present at lower density. Gray shaded regions represent 95% CI.

**Table S3: Fits for metrics with restricted age groups.**

| **Log (Indicator Density)** | | **Single Power-law** | | **Double Power-law** | | **Davies Test p-value** |
| --- | --- | --- | --- | --- | --- | --- |
| **Dependent** | **Independent** | **AIC** | **BIC** | **AIC** | **BIC** |  |
| Homicide ** | Aged 30-34 | -48.96342 | -39.08351 | -48.84127 | -32.37474 | 0.3074 |
| Flats | Aged 30-34 | 541.0798 | 552.6364 | 505.5375 | 524.7985 | 0.00 |
| Robbery ** | Aged 30-34 | -45.47834 | -33.93036 | -45.85651 | -26.60988 | 0.3334 |
| Dementia | Aged 70-74 | -647.5119 | -635.9553 | -688.1163 | -668.8553 | 0.00 |
| Dementia | Aged 85+ | -634.7232 | -623.1665 | -664.1744 | -644.9134 | 0.00 |
| Kidney Cancer | Aged 70-74 | -650.2151 | -638.6844 | -688.2069 | -688.9892 | 0.00 |
| Kidney Cancer | Aged 85+ | -558.6064 | -547.0758 | -581.8475 | -562.6298 | 0.00 |

** indicates a better fit using a single power-law opposed to a double power-law


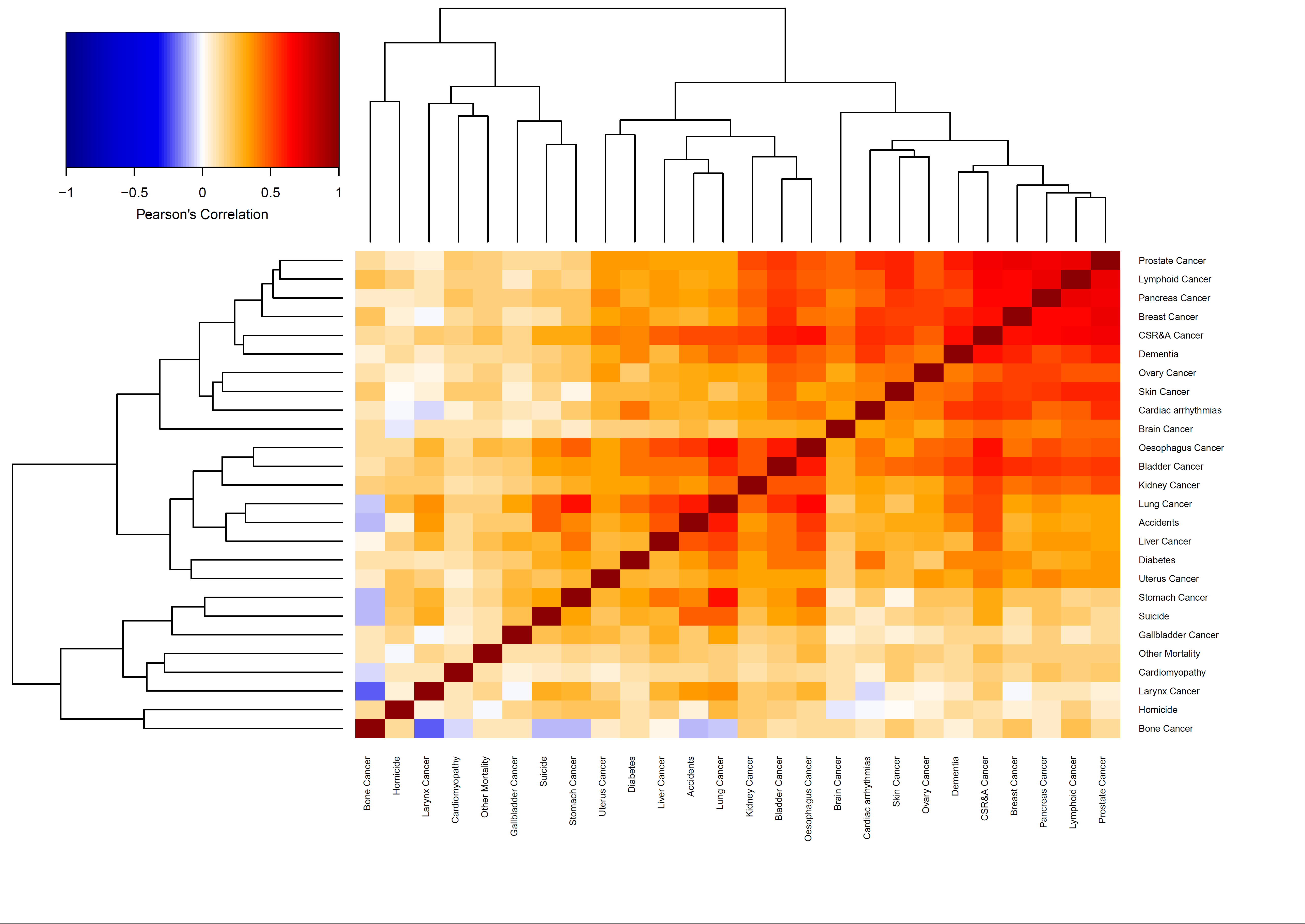


**S6 Figure. Mortality correlation heatmap in isolation.**


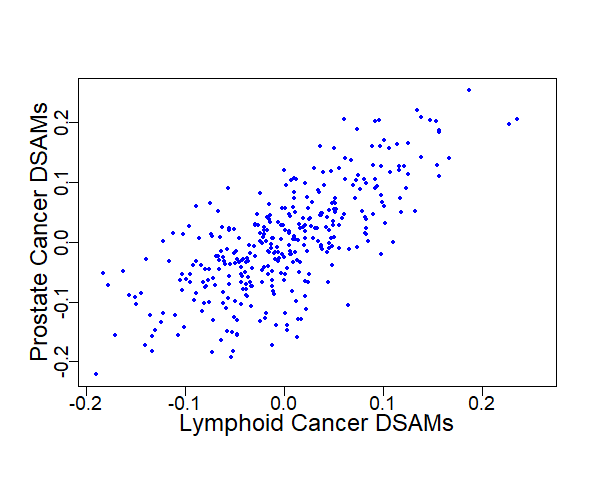


**S7 Figure. A plot of Prostate Cancer DSAMs Vs. Lymphoid Cancer DSAMs.**


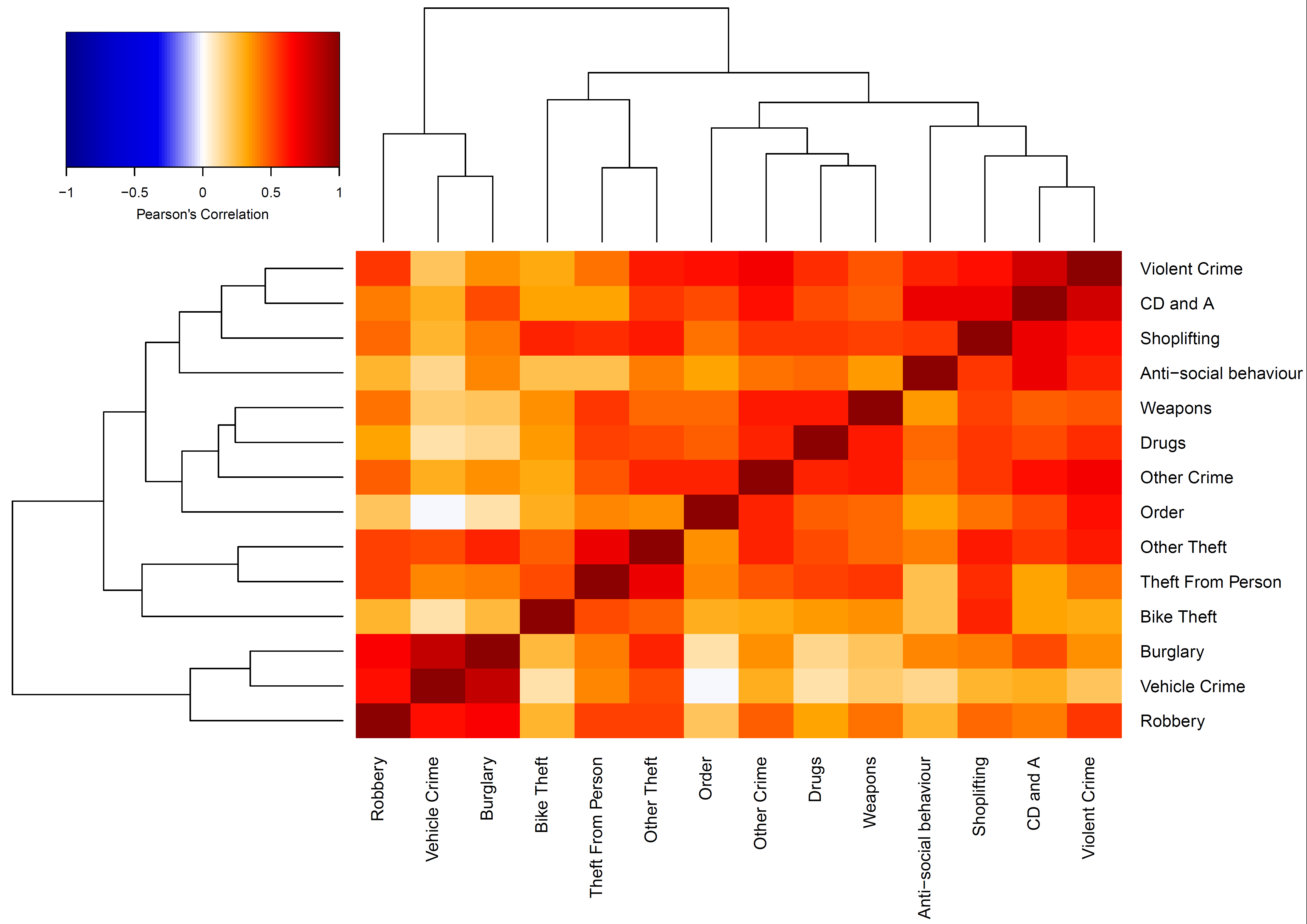


**S8 Figure. Crime correlation heatmap in isolation.**


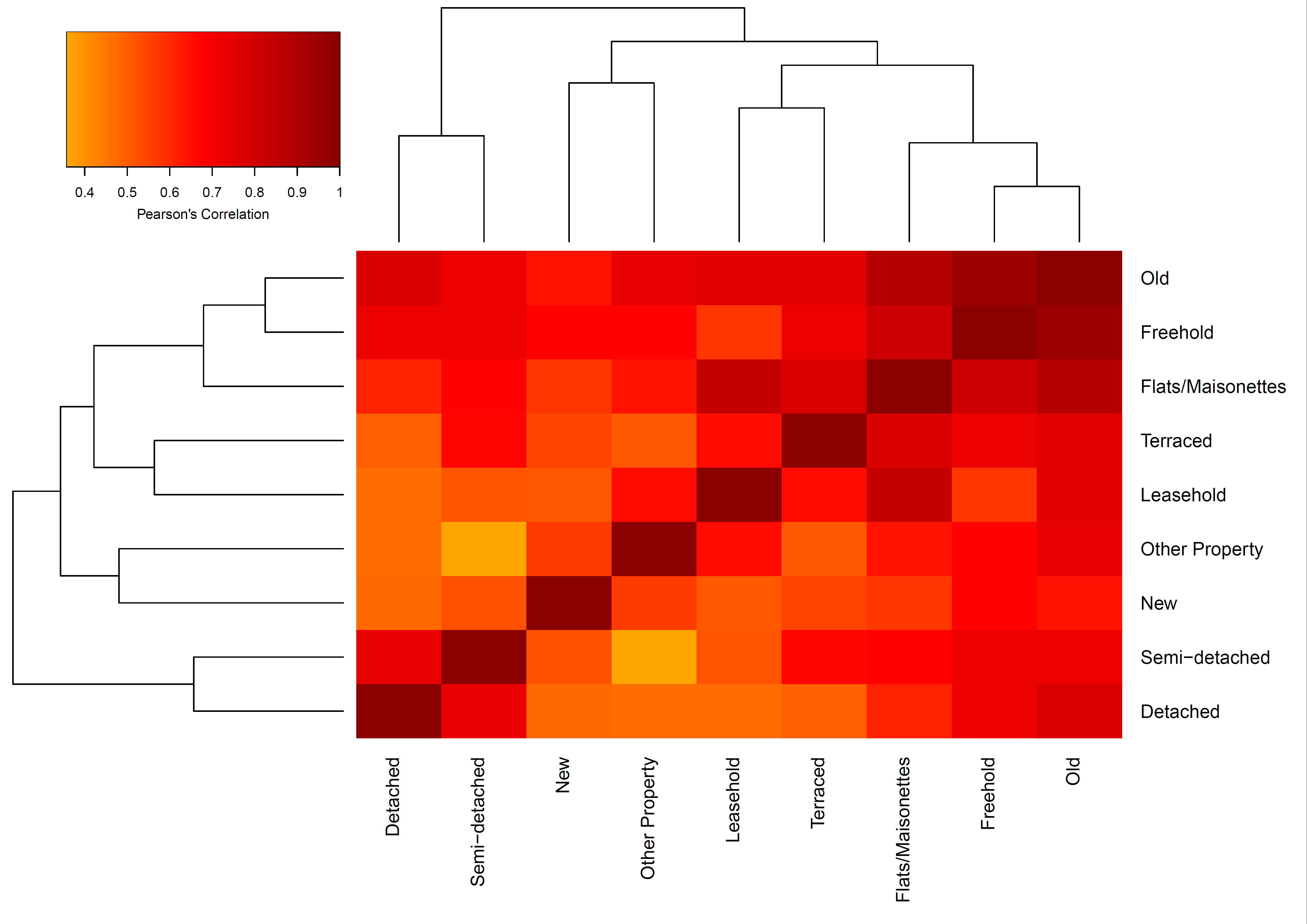


**S9 Figure. Property correlation heatmap in isolation.**


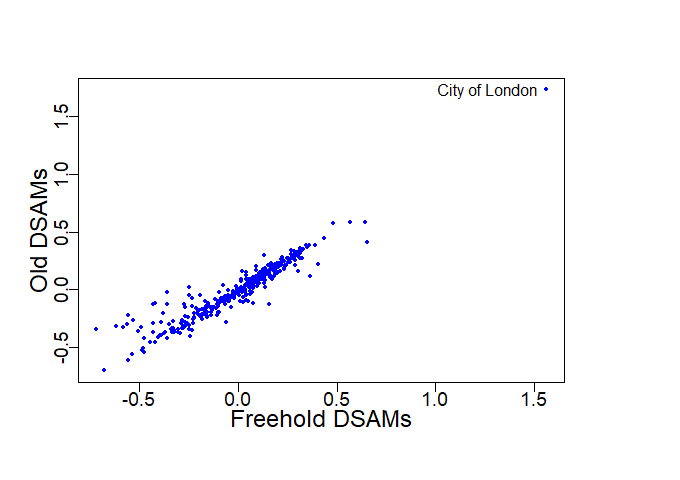


**Figure S10: Example of strong correlation between two property DSAMs.** The city of London is an extreme value, however, eliminating it from the analysis leaves a correlation of 0.938. Note property metrics are not exclusive. An old property can be a freehold property.


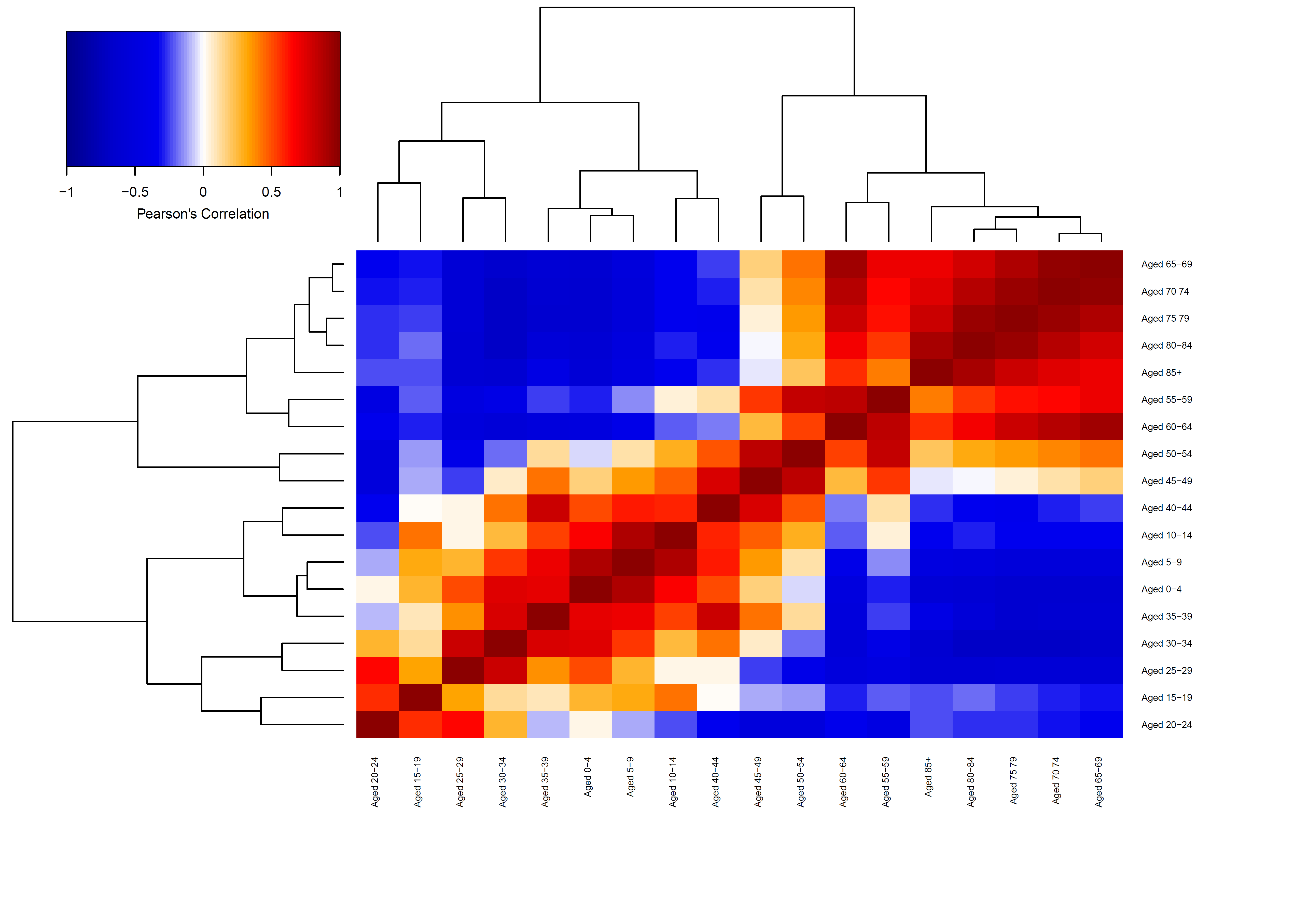


**S11 Figure. Age correlation heatmap in isolation.**


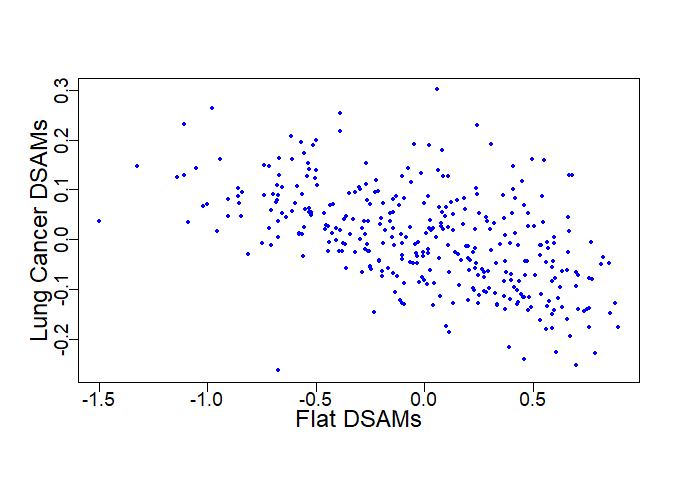


**S12 Figure. A plot of** **Flat DSAMs Vs. Lung Cancer DSAMs.**





**Figure S13. Spearman** **correlation heatmap**





**S14 Figure. Kendall correlation heatmap**


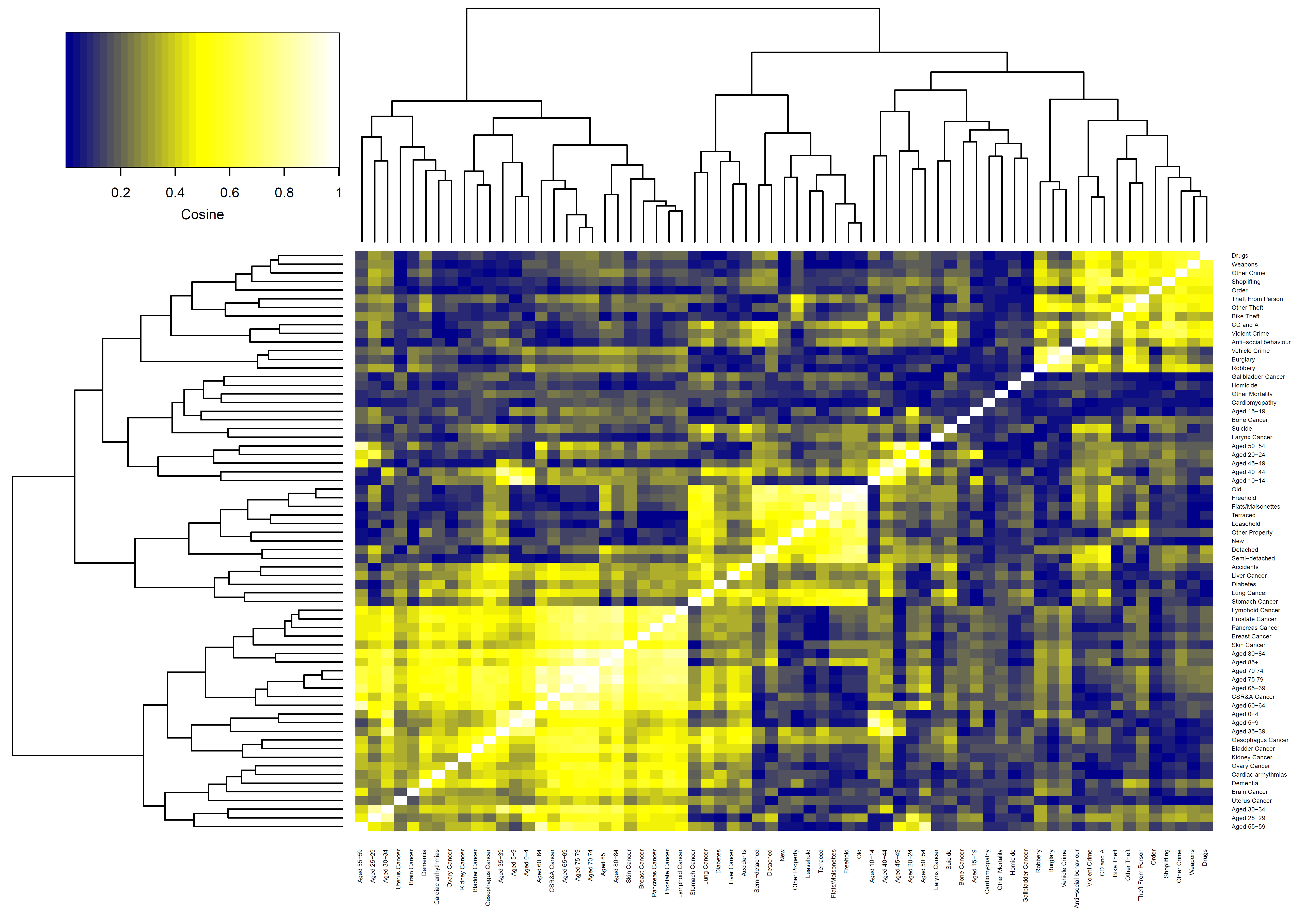


**S15 Figure. Cosine Similarity Heatmap**


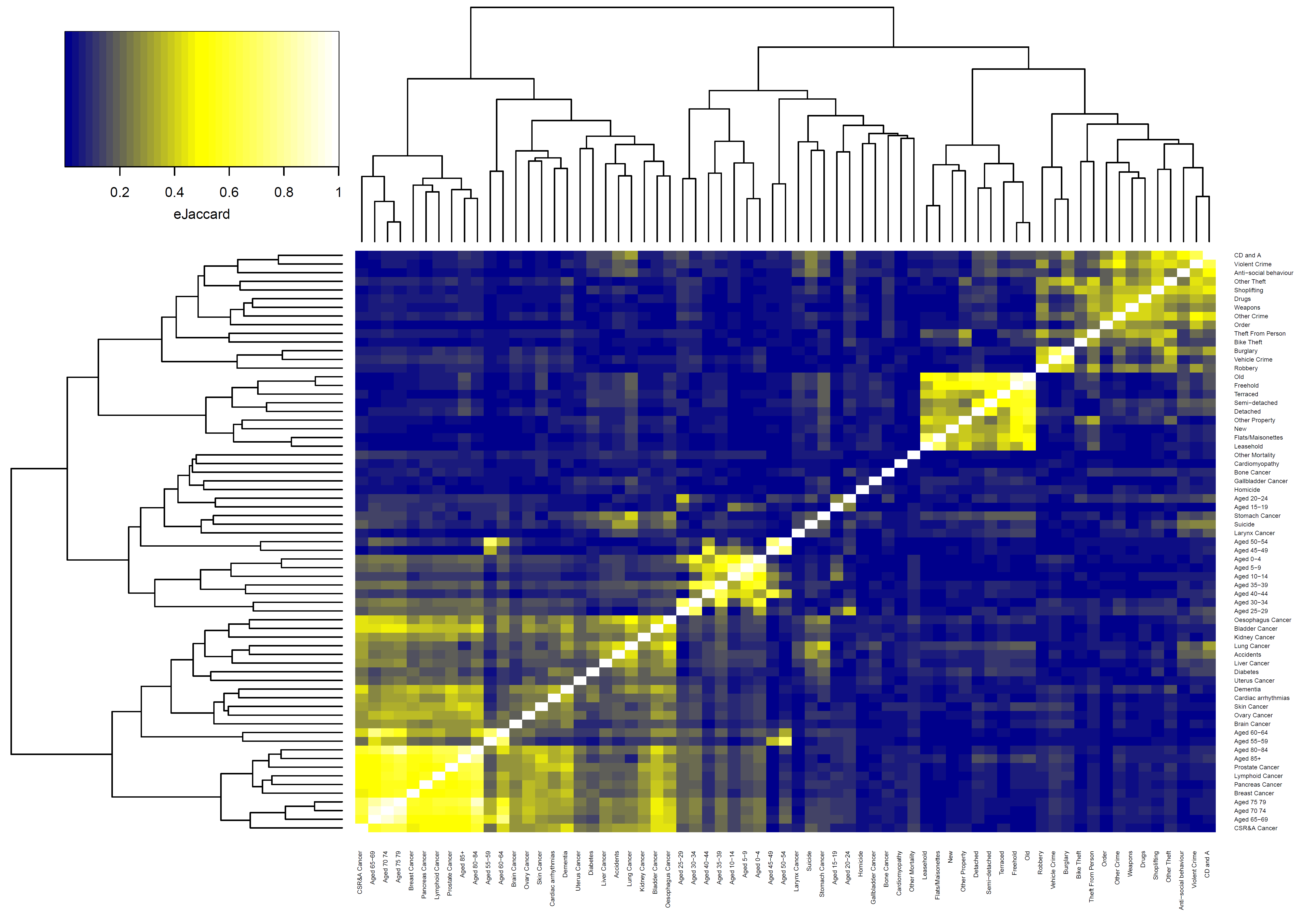


**S16 Figure. e-jaccard Similarity heatmap**

**Fig S17. Map of England and Wales.** The map of England and Wales was classified using the 2 top level clusters via the dendrogram in figure 12. The boundaries in the map are all 348 Unitary Authorities, non-metropolitan districts, metropolitan boroughs and London boroughs. The red regions belong to cluster 1 (West Sussex) and the blue regions belong to cluster 2 (Leicester). Map was created using R version (3.6.2)  (<https://www.r-project.org/>) running under R-Studio (Version 1.2.5019) (<https://rstudio.com/>).

**
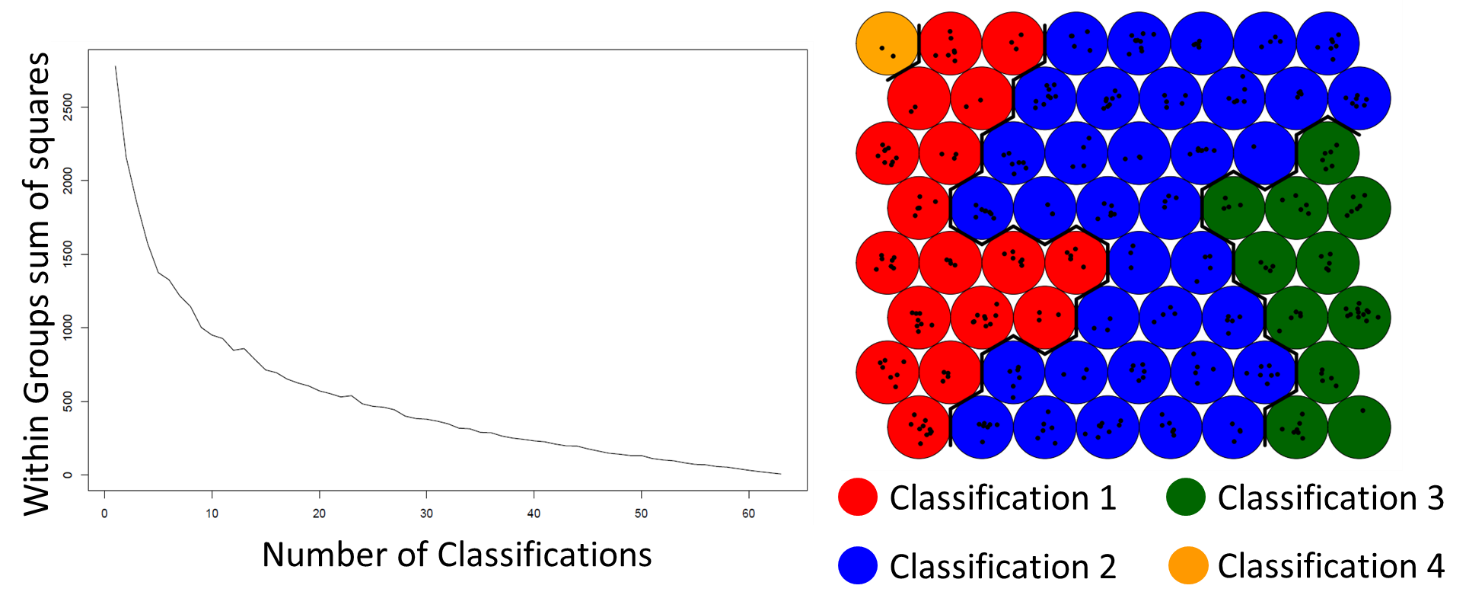
**

**Fig S18 Self-organizing map Structure.** The self-organizing map had a hexagonal 8 by 8 structure with 4 clusters chosen using the gap statistic. The nodes are orange, red, blue and green which represent clusters 1, 2, 3 and 4 respectively. The black dots within the nodes represent all 348 Unitary Authorities, non-metropolitan districts, metropolitan boroughs and London boroughs. If they are close they are similar and if they are far apart they are dissimilar in terms of their DSAMs.

| 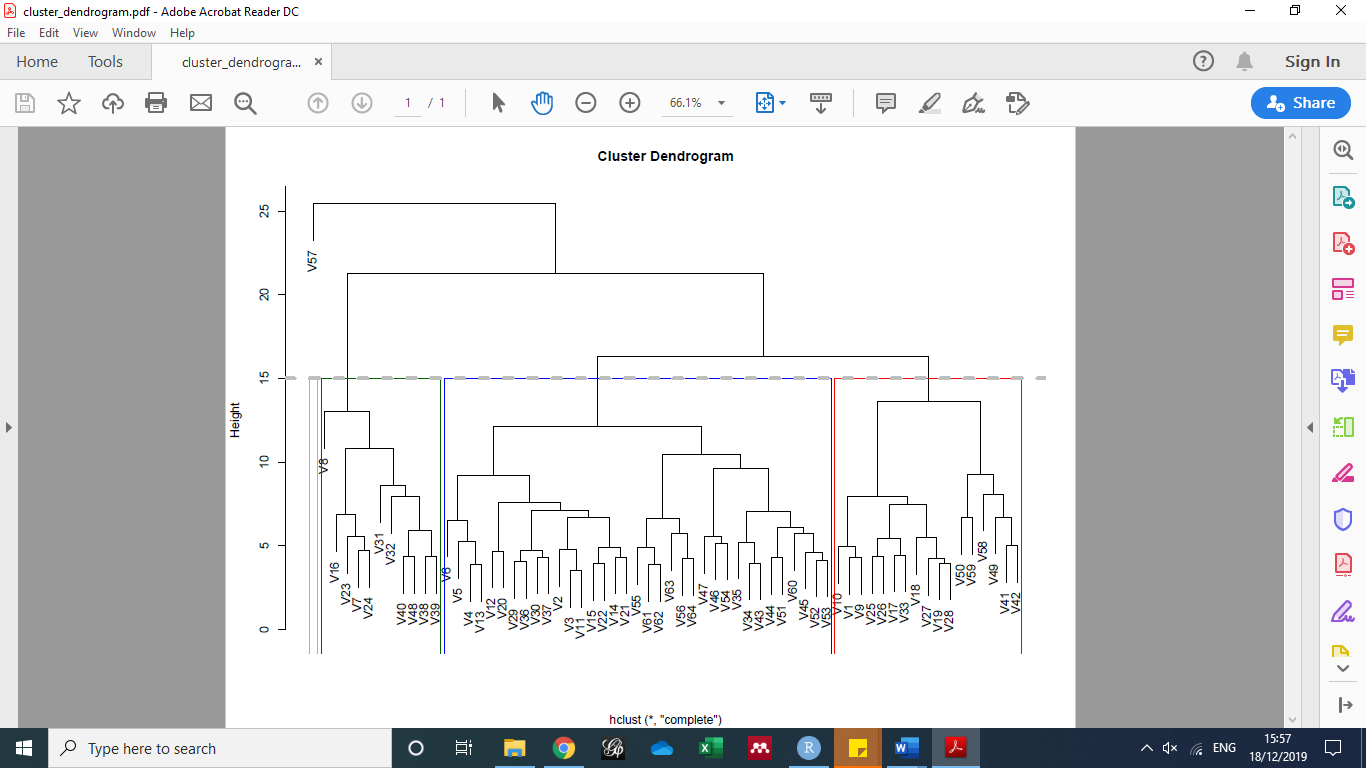 | |
| --- | --- |
| (a) | |
| 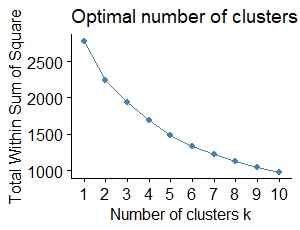 | 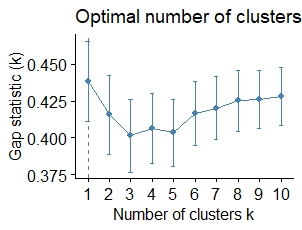 |
| (b) | (c) |

**Fig S19. Optimizing the number of clusters.** (a) A dendrogram constructed via the hierarchical clustering algorithm. The colors: orange, green, blue and red are the different clusters when $k=4$. (b) Optimal number of clusters using total within sum of squares plot for when $1\leq k\leq10$. (c) Optimal number of clusters using the gap statistic also for when $1\leq k\leq10$. Error bars represent standard errors.

| 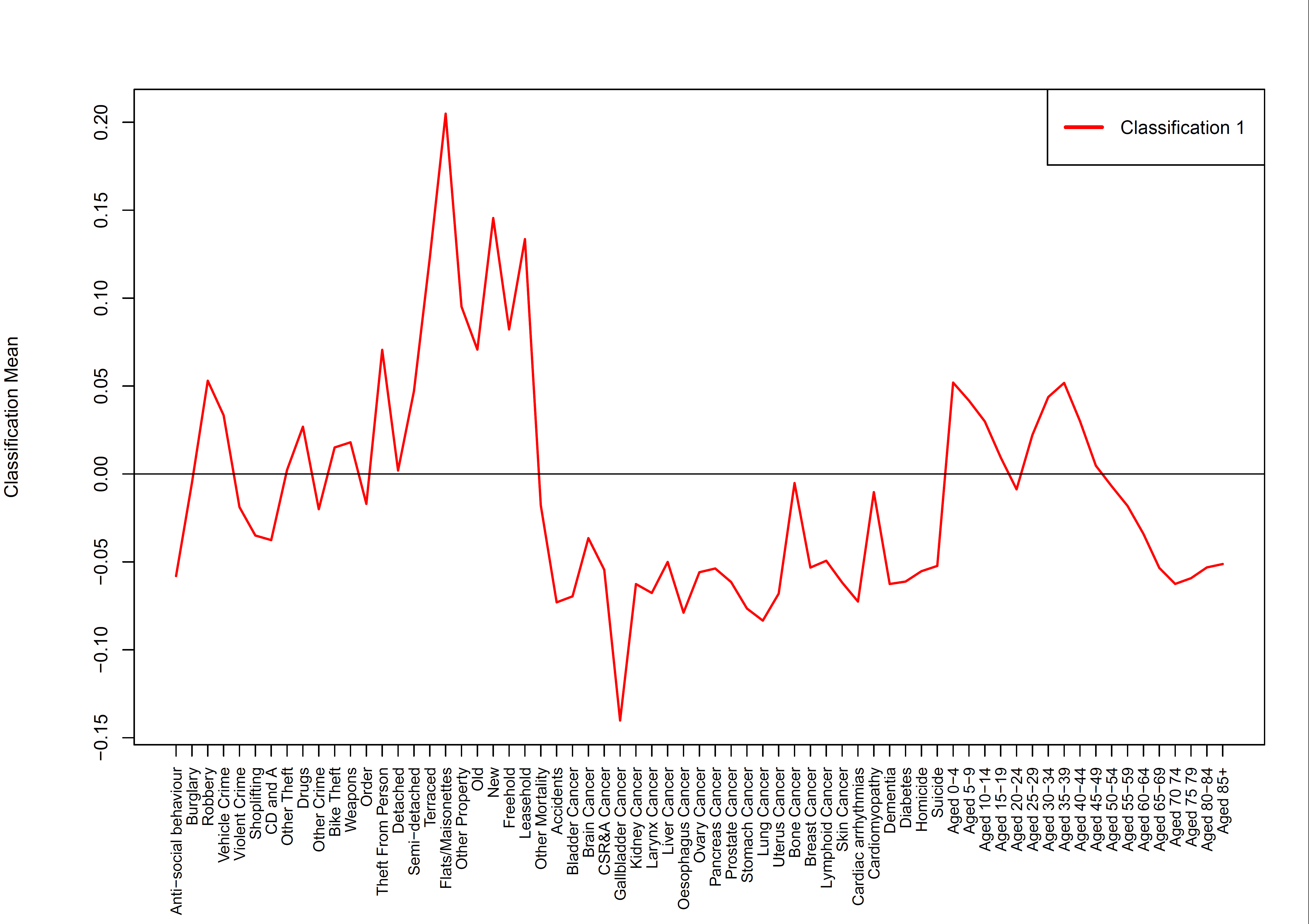 | 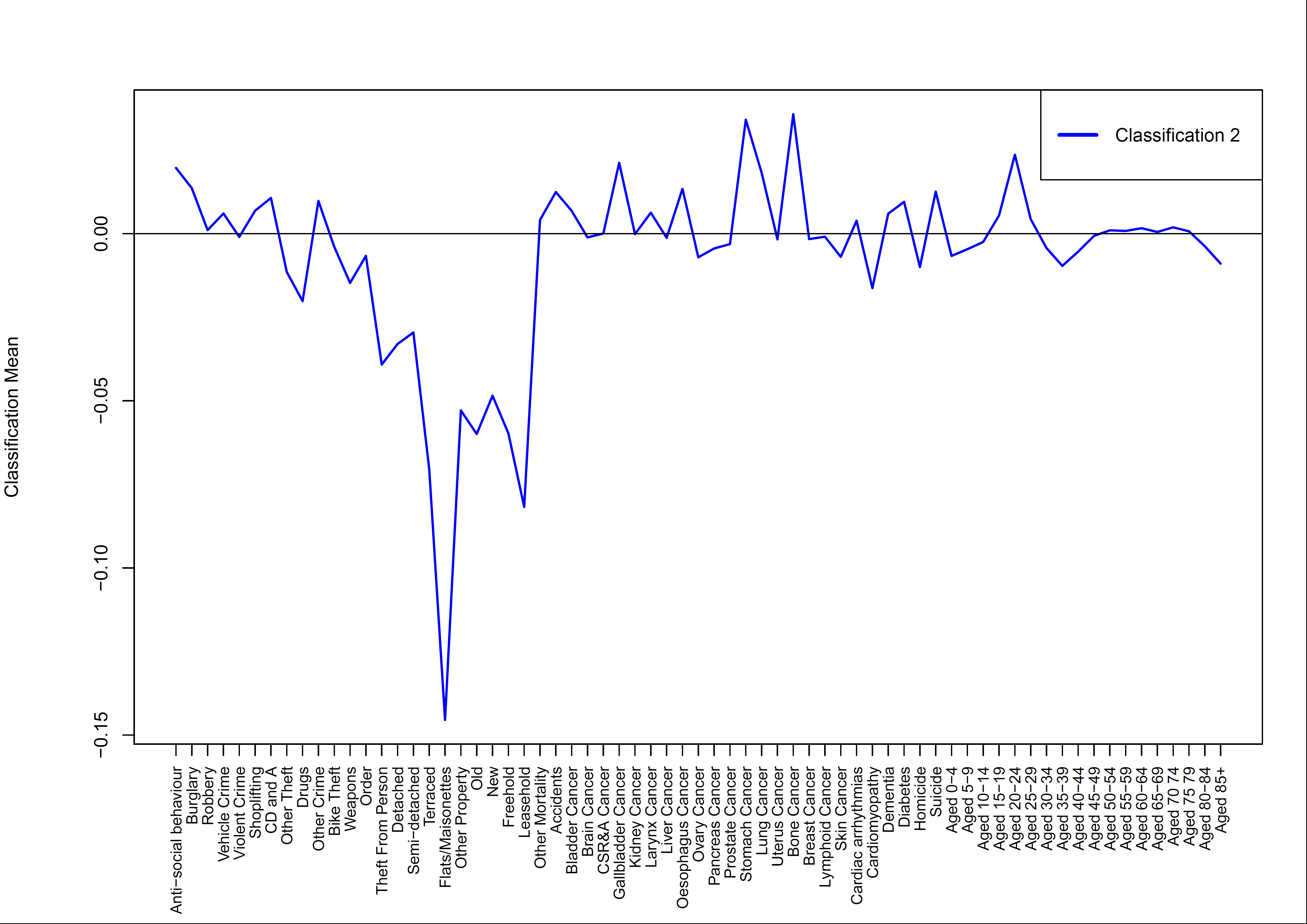 |
| --- | --- |
| (a) | (b) |
| 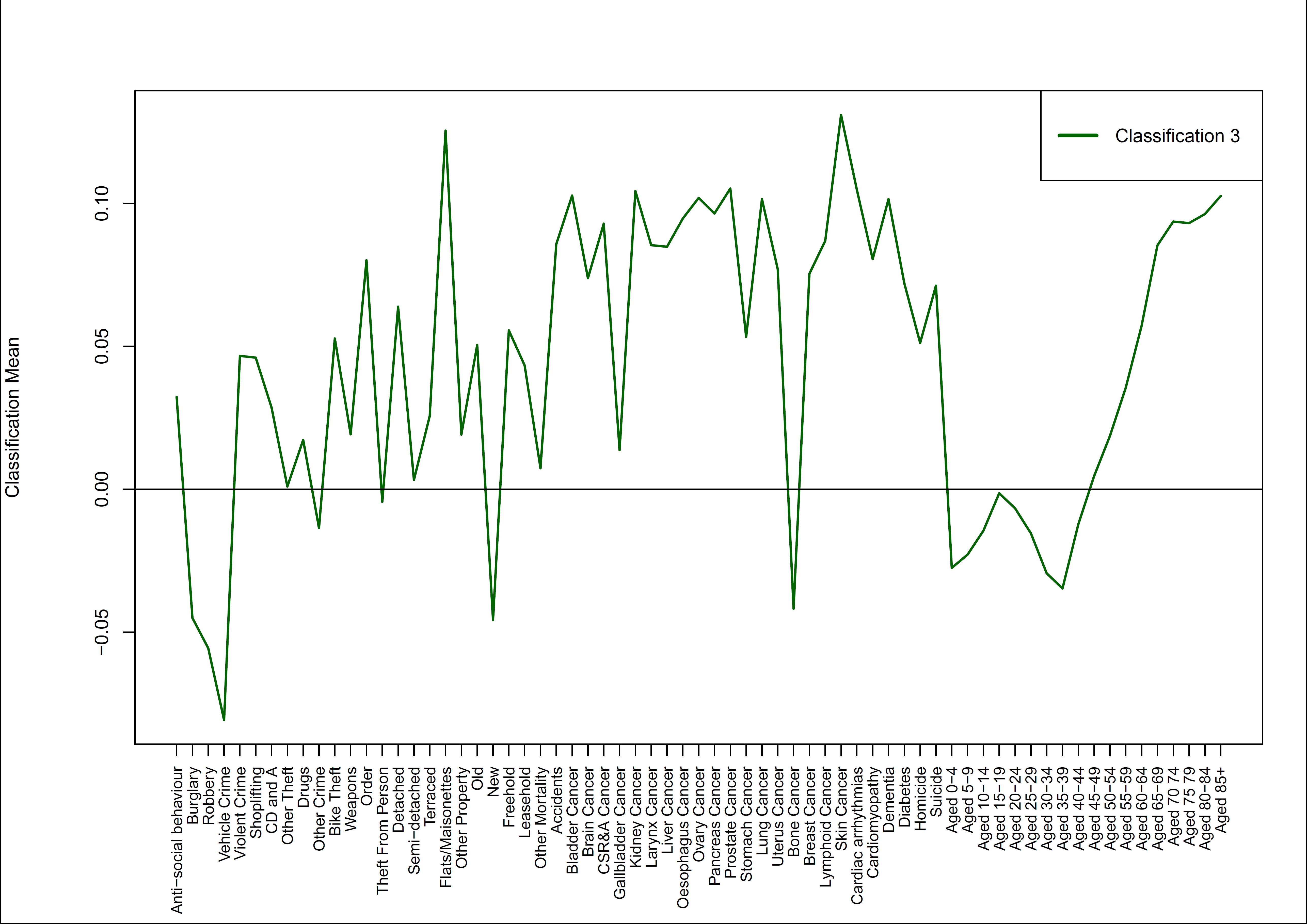 | 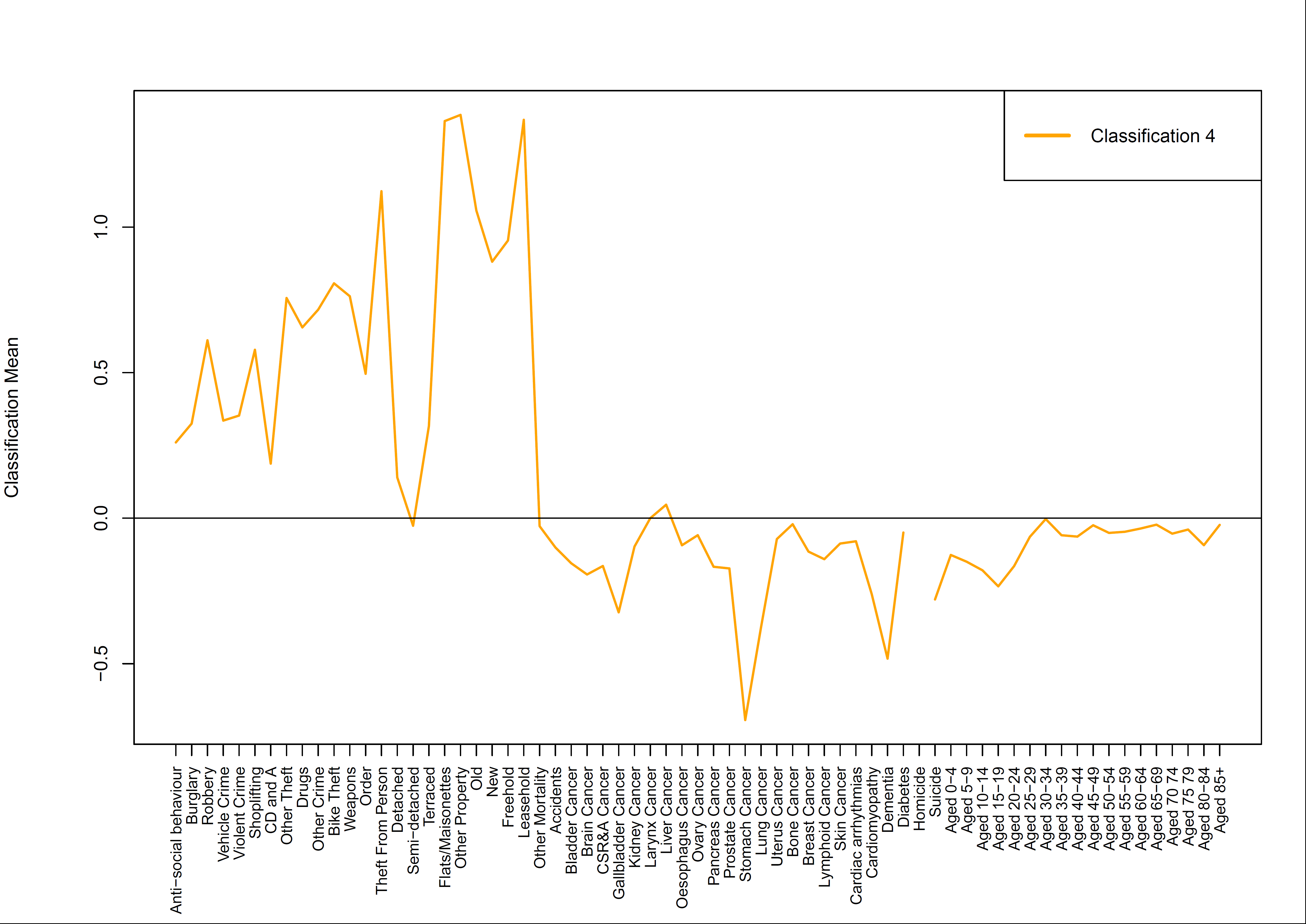 |
| (c) | (d) |

**Fig S20. Characteristics of the SOM clusters.**


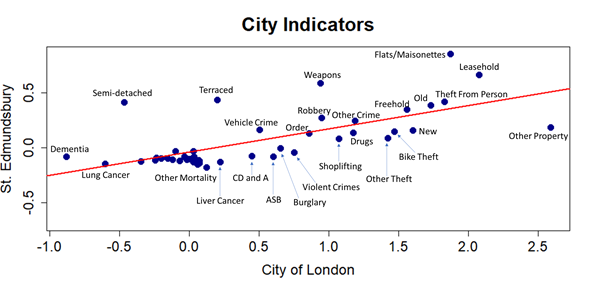


**S21 Figure. A plot of St. Edmundsbury Indicators Vs. City of London Indicators**
